# Orientation selectivity properties for the affine Gaussian derivative and the affine Gabor models for visual receptive fields

**DOI:** 10.1007/s10827-024-00888-w

**Published:** 2025-01-29

**Authors:** Tony Lindeberg

**Affiliations:** Computational Brain Science Lab, Division of Computational Science and Technology, KTH Royal Institute of Technology, SE-100 44 Stockholm, Sweden

**Keywords:** Receptive field, Orientation selectivity, Affine covariance, Simple cell, Complex cell, Vision

## Abstract

This paper presents an in-depth theoretical analysis of the orientation selectivity properties of simple cells and complex cells, that can be well modelled by the generalized Gaussian derivative model for visual receptive fields, with the purely spatial component of the receptive fields determined by oriented affine Gaussian derivatives for different orders of spatial differentiation. A detailed mathematical analysis is presented for the three different cases of either: (i) purely spatial receptive fields, (ii) space-time separable spatio-temporal receptive fields and (iii) velocity-adapted spatio-temporal receptive fields. Closed-form theoretical expressions for the orientation selectivity curves for idealized models of simple and complex cells are derived for all these main cases, and it is shown that the orientation selectivity of the receptive fields becomes more narrow, as a scale parameter ratio $\kappa$, defined as the ratio between the scale parameters in the directions perpendicular to *vs.* parallel with the preferred orientation of the receptive field, increases. It is also shown that the orientation selectivity becomes more narrow with increasing order of spatial differentiation in the underlying affine Gaussian derivative operators over the spatial domain. A corresponding theoretical orientation selectivity analysis is also presented for purely spatial receptive fields according to an affine Gabor model, showing that: (i) the orientation selectivity becomes more narrow when making the receptive fields wider in the direction perpendicular to the preferred orientation of the receptive field; while (ii) an additional degree of freedom in the affine Gabor model does, however, also strongly affect the orientation selectivity properties.

**Supplementary Information:**

The online version contains supplementary material available at 10.1007/s10827-024-00888-w.

## Introduction

The receptive fields^1^ in the primary visual cortex (V1) capture properties of the visual patterns, which are then passed on to higher layers in the visual hierarchy. Being able to understand the computational function of these receptive fields is, hence, essential for understanding the computational function of the visual system.

The task of understanding the visual system has been addressed both neurophysiologically, by measuring the response properties of neurons to visual stimuli, and by formulating mathematical models, that aim at both explaining the computational function of visual neurons, as well as enabling computational simulation of neural functions in terms of biologically plausible computer vision algorithms, or aiming at making theoretical predictions, which can then be investigated experimentally.

If we want to build computational models of the vision system, then the receptive fields of the visual neurons can be seen as the primary objects with regard to the theoretical modelling step. In terms of neurophysiological measurements, it does, however, usually imply quite a complex procedure to reconstruct the receptive fields of individual neurons. First of all, the problem of deriving a receptive field model for a visual neuron constitutes an inverse problem, which may require both an explicit model for the computational function of the neuron, as well as the need for performing a substantial number of measurements for different visual stimuli, to span a sufficiently large subspace of the variability in the possible types of image input that the neuron may be exposed to, see DeAngelis et al. (1995); DeAngelis and Anzai (2004); Ringach (2004); Sharpee (2013); Walker et al. (2019).

Mapping the orientation selectivity properties of a visual neuron, can, on the other hand, often be performed by a comparably much more straightforward procedure, by just using a probing stimulus, often a sine wave pattern, and then measuring the variations in the output of the neuron when the orientation of the stimulus is varied in the image domain (Ringach et al., 2002; Nauhaus et al., 2008; Scholl et al., 2013; Mazurek et al., 2014). For this reason, there is a substantially larger amount of available neurophysiological measurements in terms of orientation selectivity, than in terms of actually reconstructed receptive fields of visual neurons.

The purpose of this article is to present an in-depth theoretical analysis, that aims at bridging the gap between these two conceptually different ways of characterizing the properties of visual neurons, by deriving closed-form expressions for links between inherent properties of the receptive fields for simple and complex cells in the primary visual cortex and their orientation selectivity properties. In particular, we are interested in characterizing how the degree of elongation, or the eccentricity, for the receptive fields of simple cells is related to the orientation selectivity properties, for the commonly used models of simple cells in terms of either (i) an extension of the regular Gaussian derivative model (Koenderink & van Doorn, 1987, 1992; Young, 1987) into the affine Gaussian derivative model for visual receptive fields (Lindeberg, 2013, 2021), or (ii) the affine Gabor model for visual receptive fields (Marcelja, 1980; Jones & Palmer, 1987a, b; Ringach, 2002).

The motivation for studying these two models for visual receptive fields are that: (i) the generalized Gaussian derivative model can be regarded as the theoretically most principled model, by being derived by necessity in an axiomatic way from principled symmetry requirements, while (ii) the Gabor model can be regarded as the most commonly used model in the field. In addition to such idealized linear models for simple cells, we will also, assuming that complex cells can be computationally modelled as combining the output from multiple simple cells in a non-linear manner, derive corresponding relationships between properties of the receptive fields for the underlying simple cells and the orientation selectivity properties for idealized models of complex cells.

Specifically, we will focus on quantifying how the degree of orientation selectivity of a receptive field depends on how anisotropic or elongated that the underlying affine Gaussian derivatives are. We will derive explicit expressions for how the degree of orientation selectivity increases with the ratio between the scale parameters in the orientations perpendicular to *vs.* parallel with the preferred orientation of the receptive fields. In this way, we also demonstrate how closed-form theoretical analysis is possible for the generalized Gaussian derivative model for visual receptive fields, which arises from the normative theory of visual receptive fields in Lindeberg (2021). Affine Gaussian smoothing has also been earlier used for computing more accurate cues to local surface orientation, than is possible if basing the spatial smoothing operations on rotationally symmetric Gaussian kernels only (Lindeberg & Gårding, 1997).

We propose that these theoretical connections, between the orientation selectivity and the degree of elongation of the receptive fields, can be useful, when to relate the results from neurophysiological recordings of the orientation selectivity properties of visual neurons to other modelling-based functional characteristics of biological receptive fields. Specifically, in a companion paper (Lindeberg, 2024a), theoretical results derived in this paper are, in combination with properties of biological measurements of the orientation selectivity of receptive fields in the primary visual cortex by Nauhaus et al. (2008) and Goris et al. (2015), used to provide potential indirect support for a previously formulated hypothesis (Hypothesis 1 in Lindeberg 2023b Section 3.2.1). That hypothesis states that the the receptive field shapes in the primary visual cortex of higher mammals may span a variability over the eccentricity, or the degree of elongation, of the receptive fields, to support affine covariance over the population of biological receptive fields, to in turn enable the computation of more accurate cues to 3-D scene geometry, when observing the same local surface patch from different slant angles.

More generally, we propose that the theoretical results to be presented, regarding observable properties of the affine Gaussian derivative model for visual receptive fields, as well as for the affine Gabor model also studied for comparison, are important for understanding the properties of these models for visual receptive fields in the primary visual cortex. These theoretical results are also important when relating biological measurements of orientation selectivity, which have been extensively performed in the area of biological vision, to mathematical models of functional properties of the visual neurons.

Compared to the results of purely numerical simulations of properties of networks of visual neurons, the closed-form nature of the results derived in this paper for the affine Gaussian derivative model specifically makes it possible to use these results as first-class results, which can be used as primitives for inferring further properties of receptive fields based on closed-form mathematical analysis. See also (Lindeberg, 2024a) for other closed-form mathematical characterizations of properties of idealized models for visual receptive fields, based on results derived in this paper.

By performing such idealized theoretical analysis of functional properties and characteristics of visual neurons, we argue that it should be possible to reveal both inherent possibilities and limitations with regard to the computational functions of these functional primitives in the visual pathway. See also Geisler (2011) and Burge (2020) for overviews of the structurally related notion of idealized observers for visual perception. The main difference in relation to our approach, pursued according to our normative conceptual framework for visual receptive fields, is that we instead base the foundations of our analysis on symmetry properties of the environment, in a structurally similar way as symmetry properties can constitute the foundations for formulating fundamental theories in theoretical physics.

Additionally, when using a computational modelling approach to understand the function of the vision system, it is essential to also understand the theoretical properties of the model. The analysis presented in this paper shows that it is possible to fully understand the orientation selectivity properties for the affine Gaussian derivative model for simple cells in terms of the degree of elongation of the receptive fields, combined with the order of spatial differentiation. As will be shown in the paper, there is, however, not such a direct relationship between the orientation selectivity properties and the degree of elongation for the affine Gabor model as for the affine Gaussian derivative model, which is important to consider when modelling the functional properties of visual receptive fields in terms of idealized models.

Furthermore, with regard to theory developments, the results derived in this paper extend the axiomatically determined computational and normative theory for visual receptive fields in terms of the generalized Gaussian derivative model (Lindeberg, 2013, 2021) from provable covariance properties under natural image transformations (Lindeberg, 2023b, 2024b) to explicit formulations of orientation selectivity properties in closed form, for the idealized receptive fields according to this theoretically principled model for visual receptive fields.

### Structure of this article

This paper is organized as follows: After an overview of related work in Sect. 2, Sect. 3 provides the theoretical background to this work, by describing the generalized Gaussian derivative model for visual receptive fields, both in the cases of a purely spatial domain, where the receptive fields are pure affine Gaussian derivatives, and for a joint spatio-temporal domain, where the affine Gaussian derivatives are complemented by temporal derivatives of either a non-causal Gaussian kernel over the temporal domain, or a genuine time-causal kernel, referred to as the time-causal limit kernel, as well as complemented with possible velocity adaptation, to enable Galilean covariance.

Beyond these models of simple cells, we do both review a previously formulated purely spatial model for complex cells, based on a Euclidean combination of scale-normalized affine Gaussian derivatives of orders 1 and 2, as well as propose two new spatio-temporal models for complex cells, based on image measurements in terms of affine Gaussian derivatives over the spatial domain, complemented by explicit temporal processing operations. Two special cases are treated, in terms of either space-time separable spatio-temporal receptive fields, or velocity-adapted spatio-temporal receptive fields, with the latter tuned to particular motion directions and motion velocities in joint space-time.

Section 4 then performs a detailed mathematical analysis of the orientation selectivity of these models, over the three main cases of either (i) a purely spatial domain, (ii) a space-time separable spatio-temporal domain, or (iii) a velocity-adapted spatio-temporal domain. For each one of these main cases, we analyze the properties of simple cells of orders 1 and 2, corresponding to first- or second-order Gaussian derivatives, as well as the orientation selectivity properties for idealized models of complex cells, defined in terms of quasi-quadrature measures, constituting Euclidean combinations of the underlying idealized models of simple cells.

Concerning simple cells, it is shown that both the pure spatial receptive fields and the joint spatio-temporal receptive fields have similar orientation selectivity properties, which only depend on the order of spatial differentiation and the degree of anisotropy of the receptive field. For complex cells, a different dependency is, however, derived for the model based on space-time separable receptive fields, as opposed to the models for either a purely spatial domain or a joint spatio-temporal domain based on velocity-adapted spatio-temporal receptive fields.

To widen the scope of the treatment in the paper, beyond a theoretical analysis of properties of idealized visual receptive field models according to the generalized Gaussian derivative model for visual receptive fields, we will also in Sect. 5 perform a corresponding orientation selectivity analysis for an affine Gabor model of receptive fields, as well as give a derivation of affine covariant properties of a further generalized affine Gabor model, with the details of that analysis in Appendix A.2.

As a service for the reader, who may want to use the results from the theoretical analysis in this paper for comparing biological measurements of the orientation selectivity of visual neurons to characteristic properties of idealized models of visual receptive fields, we do then in Sect. 6 compute two explicit compact measures of the degree of orientation selectivity for the idealized models of receptive fields in terms of affine Gaussian derivatives, in terms of the resultant of the circular distribution of the orientation selectivity curves as well as the orientation bandwidth, as functions of the scale parameter ratio $\kappa$ of the receptive fields.

For the purpose of relating results of biological measurements performed under conditions when the angular frequency of the sine wave probe is not adapted to each stimulus orientation, as done in the main theoretical analysis in Sect. 4, we do additionally in Sect. 7 present a complementary analysis of how different variations in the choice of the angular frequency of the sine wave probe, and for spatio-temporal receptive fields also variations in the image velocity of the sine wave stimulus, affect the shapes of the resulting idealized orientation selectivity curves.

In this complementary analysis, it is specifically shown that the shapes of resulting orientation selectivity curves may be strongly dependent on the relationships between the parameters of the probing sine wave stimulus and the parameters of the receptive field, unless adapting the parameters of the sine wave probe to maximize the response of the receptive field, as done in the theoretical analysis in Sect. 4.

Finally, Sect. 8 gives a summary and discussion about some of the main results, including suggestions concerning possible extensions of the presented work.

## Related work

Hubel and Wiesel (1959, 1962, 1968, 2005) pioneered the study of visual neurons in the primary visual cortex, and introduced the taxonomy of simple and complex cells. Simple cells were simple characterized by their properties of: (i) having distinct excitatory and inhibitory regions, (ii) obeying roughly linear summation properties, (iii) the excitatory and inhibitory regions balance each other in diffuse lighting. Visual neurons that did not obey these properties were referred to as complex cells. The response of a complex cell to a visual stimulus was also reported to be much less sensitive to the position of the stimulus in the visual field than for a simple cell.

More detailed characterizations of the receptive fields of simple cells have then been performed by DeAngelis et al. (1995); DeAngelis and Anzai (2004); Ringach (2002, 2004); Conway and Livingstone (2006); Johnson et al. (2008); De and Horwitz (2021), where specifically the use of multiple white noise stimuli permit a reconstruction of the full receptive field of a visual neuron, based on theoretical results in system identification theory, assuming linearity of the neuron. More specialized methodologies to characterize the response properties of possibly non-linear visual neurons, based on using deep predictive models to generate tailored stimuli for probing and modelling the neurons, have also been been more recently developed by Walker et al. (2019).

Furthermore, more detailed characterizations of the orientation selectivity of visual neurons have been performed by Watkins and Berkley (1974); Rose and Blakemore (1974); Schiller et al. (1976); Albright (1984); Ringach et al. (2002); Nauhaus et al. (2008); Scholl et al. (2013); Sadeh and Rotter (2014); Goris et al. (2015); Li et al. (2015); Almasi et al. (2020).

While several works have been concerned with biological mechanisms for achieving orientation selectivity in the visual neurons (Somers et al., 1995; Sompolinsky & Shapley, 1997; Carandini & Ringach, 1997; Lampl et al., 2001; Ferster & Miller, 2000; Shapley et al., 2003; Seriès et al., 2004; Hansel & van Vreeswijk, 2012; Moldakarimov et al., 2014; Cogno & Mato, 2015; Priebe, 2016; Pattadkal et al., 2018; Nguyen & Freeman, 2019; Merkt et al., 2019; Wei et al., 2022; Wang et al., 2024) as well as characterizing properties of cortical maps (Bonhoeffer & Grinvald, 1991; Blasdel, 1992; Maldonado et al., 1997; Koch et al., 2016; Kremkow et al., 2016; Najafian et al., 2022; Jung et al., 2022; Fang et al., 2022; Vita et al., 2024), the focus of this work is, however, not on such specific biological implementation mechanisms, but instead on purely *functional properties of individual neurons*, that will constitute the effective results from such underlying computational mechanisms between different neurons.

A detailed study of how the orientation selective of neurons in the primary visual cortex can be modulated for cats that wear permanently mounted googles, that alter the directional distribution of the incoming visual stimuli, has been presented by Sasaki et al. (2015), showing that long exposure to such stimuli affects the elongation of receptive fields, and how such elongation affects the orientational selectivity.

Mathematical models of simple cells have been formulated, in terms of Gabor filters (Marcelja, 1980; Jones & Palmer, 1987a, b; Porat & Zeevi, 1988) or Gaussian derivatives (Koenderink, 1984; Koenderink & van Doorn, 1987, 1992; Young, 1987; Young et al., 2001; Young & Lesperance, 2001; Lindeberg, 2013, 2021). Specifically, theoretical models of early visual processes in terms of Gaussian derivatives have been formulated or used in Lowe (2000); May and Georgeson (2007); Hesse and Georgeson (2005); Georgeson et al. (2007); Hansen and Neumann (2008); Wallis and Georgeson (2009); Wang and Spratling (2016); Pei et al. (2016); Ghodrati et al. (2017); Kristensen and Sandberg (2021); Abballe and Asari (2022); Ruslim et al. (2023); Wendt and Faul (2024).

Beyond the work by Hubel and Wiesel, the properties of complex cells have been further characterized by Movshon et al. (1978); Emerson et al. (1987); Martinez and Alonso (2001); Touryan et al. (2002, 2005); Rust et al. (2005); van Kleef et al. (2010); Goris et al. (2015); Li et al. (2015); Almasi et al. (2020), as well as modelled computationally by Adelson and Bergen (1985); Heeger (1992); Serre and Riesenhuber (2004); Einhäuser et al. (2002); Kording et al. (2004); Merolla and Boahn (2004); Berkes and Wiskott (2005); Carandini (2006); Hansard and Horaud (2011); Franciosini et al. (2019); Lian et al. (2021); Oleskiw et al. (2024); Yedjour and Yedjour (2024).

The notion of affine Gaussian smoothing, with its associated notion of affine Gaussian derivatives, was derived axiomatically in Lindeberg (2011) and was proposed as a spatial model of simple cells in Lindeberg (2013, 2021). This model has also been extended to complex cells in (Lindeberg 2020 Section 5). Parallel extensions of the affine Gaussian derivative model for simple cells to spatio-temporal receptive fields have been performed in Lindeberg (2016, 2021).

## Receptive field models based on affine Gaussian derivatives

For modelling the response properties of simple cells, we will in this paper make use of the affine Gaussian derivative model for linear receptive fields, which has been theoretically derived in a principled axiomatic manner in Lindeberg (2011), and then been demonstrated in Lindeberg (2013, 2021) to well model the spatial properties of simple cells in the primary cortex, as established by neurophysiological measurements by DeAngelis et al. (1995); DeAngelis and Anzai (2004); Johnson et al. (2008); see Figures 16–17 in Lindeberg (2021) for comparisons between biological receptive fields and computational models in terms of affine Gaussian derivatives, as will be used as a basis for the analysis in this paper.

In this section, we shall review main components of this theory, which we shall then build upon in the theoretical analysis in Sect. 4, concerning the orientation selectivity properties of these receptive field models, based on the affine Gaussian derivative model for simple cells over a purely spatial domain, as well as a corresponding generalized affine Gaussian derivative model for simple cells over a joint spatio-temporal domain. Based on these models of simple cells, we shall also define models for complex cells, both over a purely spatial image domain and over a joint spatio-temporal domain, where the joint spatio-temporal models are new. Since the analysis to be performed in Sect. 4 will make use of very specific properties of this receptive field model, while this model may be less known than the Gabor model for visual receptive fields, this review will be rather detailed, so as to be sufficiently self-contained, to support the theoretical analysis that will be later presented in Sect. 4.

### Purely spatial model of simple cells

If we initially disregard the temporal dependencies of the simple cells, we can formulate a purely spatial model of linear receptive fields with orientation preference according to (Lindeberg 2021 Equation (23)), see (Lindeberg 2021 Figure 7) for illustrations of such receptive fields;

1 $$\begin{aligned} \begin{aligned}&T_{\text{ simple }}(x_1, x_2;\; \sigma _{\varphi }, \varphi , \varSigma _{\varphi }, m) \\&= T_{\varphi ^m,\text{ norm }}(x_1, x_2;\; \sigma _{\varphi }, \varSigma _{\varphi }) = \sigma _{\varphi }^{m} \, \partial _{\varphi }^{m} \left( g(x_1, x_2;\; \varSigma _{\varphi }) \right) , \end{aligned} \end{aligned}$$

where

- $\varphi$ represents the preferred orientation of the receptive field,
- $\sigma _{\varphi }$ represents the amount of spatial smoothing^2^ in the direction $\varphi$ (in units of the spatial standard deviation^3^),
- $\partial _{\varphi }^m = (\cos \varphi \, \partial _{x_1} + \sin \varphi \, \partial _{x_2})^m$ is an *m*:th-order directional derivative operator^4^ in the direction $\varphi$,
- $\varSigma _{\varphi }$ is a symmetric positive definite covariance matrix, with one of its eigenvectors aligned with the direction of $\varphi$, and
- $g(x;\; \varSigma _{\varphi })$ is a 2-D affine Gaussian kernel with its shape determined by the covariance matrix $\varSigma _{\varphi }$
  2 $$\begin{aligned} g(x;\; \varSigma _{\varphi }) = \frac{1}{2 \pi \sqrt{\det \varSigma _{\varphi }}} e^{-x^T \varSigma _{\varphi }^{-1} x/2} \end{aligned}$$
  for $x = (x_1, x_2)^T$, and with one of the eigenvectors of $\varSigma _{\varphi }$ parallel to the orientation $\varphi$.

For $m = 1$ and $m = 2$, differentiation of the affine Gaussian kernel and introducing the following parameterization of the spatial covariance matrix

3 $$\begin{aligned} \varSigma _{\varphi } = \left( \begin{array}{cc} C_{11} & C_{12} \\ C_{12} & C_{22} \end{array} \right) \end{aligned}$$

with

4 $$\begin{aligned} {\begin{matrix} C_{11}&= \sigma _1^2 \, \cos ^2 \varphi + \sigma _2^2 \, \sin ^2 \varphi , \end{matrix}}\end{aligned}$$

5 $$\begin{aligned} {\begin{matrix} C_{12}&= (\sigma _1^2 - \sigma _2^2) \, \cos \varphi \, \sin \varphi , \end{matrix}}\end{aligned}$$

6 $$\begin{aligned} {\begin{matrix} C_{22}&= \sigma _1^2 \, \sin ^2 \varphi + \sigma _2^2 \, \cos ^2 \varphi , \end{matrix}} \end{aligned}$$

as well as reformulating the arguments with respect to this more explicit parameterization, leads to the following explicit expressions, for an arbitrary preferred orientation $\varphi$:

7 $$\begin{aligned} {\begin{matrix} T_{\text{ simple }}(x_1, x_2;\; \sigma _1, \sigma _2, \varphi , 1) = \end{matrix}}\nonumber \\ {\begin{matrix} = \sigma _1 \, (\cos (\varphi ) \, \partial _{x_1} + \sin (\varphi ) \, \partial _{x_2}) \, g(x_1, x_2;\; \varSigma _{\varphi }) \end{matrix}}\nonumber \\ {\begin{matrix} = -\frac{(x_1 \cos (\varphi )+x_2 \sin (\varphi ))}{2 \pi \, \sigma _1^2 \, \sigma _2} \times \end{matrix}}\nonumber \\ {\begin{matrix} e^{ -\frac{\left( \sigma _1^2+\sigma _2^2\right) \left( x_1^2+x_2^2\right) -(\sigma _1-\sigma _2) (\sigma _1+\sigma _2) (2 x_1 x_2 \sin (2 \varphi )+\cos (2 \varphi ) (x_1-x_2) (x_1+x_2))}{4 \sigma _1^2 \sigma _2^2}} \end{matrix}}\end{aligned}$$

8 $$\begin{aligned} {\begin{matrix} T_{\text{ simple }}(x_1, x_2;\; \sigma _1, \sigma _2, \varphi , 2) = \end{matrix}}\nonumber \\ {\begin{matrix} =\! \sigma _1^2 \, (\cos ^2 (\varphi ) \, \partial _{x_1 x_1} +\! 2 \cos (\varphi ) \sin (\varphi ) \, \partial _{x_1 x_2} + \sin ^2(\varphi ) \, \partial _{x_2 x_2}) \, \end{matrix}}\nonumber \\ {\begin{matrix} \quad \quad g(x_1, x_2;\; \varSigma _{\varphi }) \end{matrix}}\nonumber \\ {\begin{matrix} =\! \frac{ \left( x_1^2+x_2^2 -2 \sigma _1^2 + \cos (2 \varphi ) \left( x_1^2-x_2^2\right) +2 x_1 x_2 \sin (2 \varphi ) \right) }{4 \pi \, \sigma _1^3 \, \sigma _2} \!\times \! \end{matrix}}\nonumber \\ {\begin{matrix} e^{ -\frac{\left( \sigma _1^2+\sigma _2^2\right) \left( x_1^2+x_2^2\right) -(\sigma _1-\sigma _2) (\sigma _1+\sigma _2) (2 x_1 x_2 \sin (2 \varphi )+\cos (2 \varphi ) (x_1-x_2) (x_1+x_2))}{4 \sigma _1^2 \sigma _2^2}}. \end{matrix}} \end{aligned}$$

In the above expressions for the spatial receptive field model $T_{\text{ simple }}$, the multiplication of the *m*:th-order directional derivative operator in the direction $\varphi$ by the spatial scale parameter $\sigma _{\varphi }$ in the same direction, implements scale-normalized derivatives Lindeberg (1998) according to

9 $$\begin{aligned} \partial _{x_1^{\alpha _1} x_2^{\alpha _2},\text{ norm }} = \sigma ^{\gamma (\alpha _1 + \alpha _2)} \, \partial _{x_1^{\alpha _1} x_2^{\alpha _2}}, \end{aligned}$$

where we here, for simplicity, choose the scale normalization power $\gamma = 1$ to simplify the following calculations.

This notion of spatial scale-normalized derivatives implies that we measure the amplitude of local spatial variations with respect to a given scale level, and makes it possible to define scale-invariant feature responses, that assume the same magnitude for input image structures of different spatial extent, provided that the spatial scale levels are adapted to the characteristic length of spatial variations in the image data. Specifically, this spatial scale normalization of the spatial receptive field response implies scale selective properties in the sense that the receptive field will produce its maximum response over spatial scales at a spatial scale proportional to a characteristic length in the image data.

Note that this model for the spatial dependency of simple cells, in terms of affine Gaussian derivatives, goes beyond the previous biological modelling results by Young (1987), in turn with very close relations to theoretical modelling results by Koenderink and van Doorn (1987, 1992), in that the spatial smoothing part of the receptive field is here spatially anisotropic, and thereby allows for higher orientation selectivity compared to defining (regular) Gaussian derivatives from partial derivatives or directional derivatives of rotationally symmetric Gaussian kernels, as used by Young and Koenderink and van Doorn. Direct comparisons with biological receptive fields, see Figures 16–17 in Lindeberg (2021), also show that biological simple cells are more anisotropic than can be well modelled by directional derivatives of rotationally symmetric Gaussian kernels.

### Purely spatial model of complex cells

In (Lindeberg 2020 Section 5) it was proposed that some of the qualitative properties of complex cells, of being both (i) polarity-independent, (ii) approximately phase-independent and (ii) not obeying a superposition principle, as opposed to polarity-dependent as well as strongly phase-dependent, as simple cells are, can modelled by combining first- and second-order directional affine Gaussian derivative responses of the form,^5^

10 $$\begin{aligned} \mathcal{Q}_{\varphi ,\text{ spat },\text{ norm }} L = \sqrt{\frac{L_{\varphi ,\text{ norm }}^2 + C_{\varphi } \, L_{\varphi \varphi ,\text{ norm }}^2}{\sigma _{\varphi }^{2\varGamma }}}, \end{aligned}$$

where

- $L_{\varphi ,\text{ norm }}$ and $L_{\varphi \varphi ,\text{ norm }}$ represent the results of applying scale-normalized directional affine Gaussian derivative operators of orders 1 and 2, respectively, according to Eq. (1), to the input image *f*:
  11 $$\begin{aligned} L_{\varphi ,\text{ norm }}(x_1, x_2;\; \sigma _{\varphi }, \varSigma _{\varphi }) =\\ = T_{\varphi ,\text{ norm }}(x_1, x_2;\; \sigma _{\varphi }, \varSigma _{\varphi }) * f(x_1, x_2), \end{aligned}$$
  12 $$\begin{aligned} L_{\varphi \varphi ,\text{ norm }}(x_1, x_2;\; \sigma _{\varphi }, \varSigma _{\varphi }) =\\ = T_{\varphi \varphi ,\text{ norm }}(x_1, x_2;\; \sigma _{\varphi }, \varSigma _{\varphi }) * f(x_1, x_2), \end{aligned}$$
- $C_{\varphi } > 0$ is a weighting factor between first and second-order information, and
- $\varGamma \ge 0$ is a complementary scale normalization parameter, that we, however, henceforth will set to zero, to simplify the following treatment.

This model can be seen as an affine Gaussian derivative analogue of the energy model of complex cells proposed by Adelson and Bergen (1985) and Heeger (1992), specifically the fact that receptive fields similar to first- *vs.* second-order derivatives are reported to occur in pairs (Valois et al., 2000), resembling properties of approximate quadrature pairs, as related by a Hilbert transform (Bracewell 1999, pp. 267–272). The model is also closely related to the proposal by Koenderink and van Doorn (1990) to sum up the squares of first- and second-order derivative response, in a corresponding way as cosine wave and sine wave responses are combined in a Euclidean manner, to get a more phase-independent response.

For a perfect quadrature pair of filters, the sum of the squares of the filter responses will be spatially constant for any sine wave of any frequency and phase. The quasi-quadrature entity will instead have the property that the response will be spatially constant, or alternatively have only relatively moderate ripples, at or near the spatial scale level at which the quasi-quadrature measure assumes its maximum value over spatial scales, provided that the value of the weighting parameter $C_{\varphi }$ is properly chosen (see Lindeberg 1998 Figure 21 and Lindeberg 2018 Equation (26)).

In this way, the quasi-quadrature measure combines the responses of the first- and second-order affine Gaussian derivative operators in a complementary manner, where the first-order derivatives correspond to odd filters (antisymmetric under reflection) and the second-order derivatives to even filters (symmetric under reflection).

### Joint spatio-temporal models of simple cells

For modelling the joint spatio-temporal behaviour of linear receptive fields with orientation preference, in (Lindeberg 2021 Section 3.2) the following model is derived from theoretical arguments, see (Lindeberg 2021 Figures 10-11 for illustrations)

13 $$\begin{aligned} {\begin{matrix} T_{\text{ simple }}(x_1, x_2, t;\; \sigma _{\varphi }, \sigma _t, \varphi , v, \varSigma _{\varphi }, m, n) \end{matrix}}\nonumber \\ {\begin{matrix}&= T_{{\varphi }^m, {\bar{t}}^n,\text{ norm }}(x_1, x_2, t;\; \sigma _{\varphi }, \sigma _t, v, \varSigma _{\varphi }) \end{matrix}}\nonumber \\ {\begin{matrix}&= \sigma _{\varphi }^{m} \, \sigma _t^{n} \, \partial _{\varphi }^{m} \,\partial _{\bar{t}}^n \left( g(x_1 - v_1 t, x_2 - v_2 t;\; \varSigma _{\varphi }) \, h(t;\; \sigma _t) \right) , \end{matrix}} \end{aligned}$$

where (for symbols not previously defined in connection with Eq. (1))

- $\sigma _t$ represents the amount of temporal smoothing (in units of the temporal standard deviation),
- $v = (v_1, v_2)^T$ represents a local motion vector, in the direction $\varphi$ of the spatial orientation of the receptive field,
- $\partial _{\bar{t}}^n = (\partial _t + v_1 \, \partial _{x_1} + v_2 \, \partial _{x_2})^n$ represents an *n*:th-order velocity-adapted temporal derivative operator,
- $h(t;\; \sigma _t)$ represents a temporal smoothing kernel with temporal standard deviation $\sigma _t$.

In the case of non-causal time (where the future can be accessed), the temporal kernel can be determined to be a 1-D Gaussian kernel

14 $$\begin{aligned} h(t;\; \sigma _t) = \frac{1}{\sqrt{2 \pi } \sigma _t} e^{-t^2/2\sigma _t^2}, \end{aligned}$$

whereas in the case of time-causal time (where the future cannot be accessed), the temporal kernel can instead be chosen as the time-causal limit kernel (Lindeberg 2016 Section 5, 2023a Section 3)

15 $$\begin{aligned} h(t;\; \sigma _t) = \psi (t;\; \sigma _t, c), \end{aligned}$$

defined by having a Fourier transform of the form

16 $$\begin{aligned} \hat{\Psi }(\omega ;\; \sigma _t, c) = \prod _{k=1}^{\infty } \frac{1}{1 + i \, c^{-k} \sqrt{c^2-1} \, \sigma _t \, \omega }, \end{aligned}$$

and corresponding to an infinite set of first-order integrators coupled in cascade with specifically chosen time constants to enable temporal scale covariance, where the distribution parameter $c > 1$ describes the ratio between adjacent discrete temporal scale levels in this temporal scale-space model.

In analogy with the spatial scale normalization in the previous purely spatial model, multiplication of the *n*:th-order velocity-adapted temporal derivative operator $\partial _{\bar{t}}^n$ by the temporal standard deviation $\sigma _t$ raised to the power of *n* implements scale-normalized velocity-adapted temporal derivatives according to

17 $$\begin{aligned} \partial _{\bar{t},\text{ norm }}^n = \sigma _t^{\gamma n} \, \partial _{\bar{t}}^n, \end{aligned}$$

as an extension of Eq. (9) from the spatial to the temporal domain, see Lindeberg (2017). Also here, for simplicity, we restrict ourselves to the specific choice of the scale normalization parameter $\gamma = 1$. By this temporal scale normalization, the temporal component of the spatio-temporal receptive field will have scale selective properties, implying that it will produce its maximum response over temporal scales at a temporal scale proportional to a characteristic temporal duration of the temporal structures in the video data.

In Figure 18 in Lindeberg (2021), it is demonstrated that this model well captures the qualitative properties of simple cells in the primary visual cortex, as established by neurophysiological cell recordings by DeAngelis et al. (1995); DeAngelis and Anzai (2004), regarding both space-time separable receptive fields and velocity-adapted receptive fields, tuned to particular motion directions in joint space-time.

Note that these spatio-temporal models of simple cells go beyond the previous biological modelling results by Young et al. (2001); Young and Lesperance (2001) in that: (i) the purely spatial smoothing component of the receptive field model is based on anisotropic Gaussian kernels as opposed to rotationally symmetric Gaussian kernels, (ii) this model also incorporates a truly time-causal model, that takes into explicit account that the future cannot be accessed in a real-world situation, and (iii) the parameterization of the spatio-temporal filter shapes is different, and more closely aligned to the inherent geometry of the imaging situation.

### Joint spatio-temporal models of complex cells

#### Model based on space-time separable receptive fields

For modelling qualitative properties of complex cells over the joint spatio-temporal domain, we can extend the spatial quasi-quadrature measure in Eq. (10) to the following spatio-temporal quasi-quadrature measure, that operates on a combination of spatial directional derivatives and temporal derivatives according to

18 $$\begin{aligned} (\mathcal{Q}_{\varphi ,\text{ sep },\text{ norm }} L)^2 = \left( \left( L_{\varphi ,t,\text{ norm }}^2 + C_t \, L_{\varphi ,tt,\text{ norm }}^2 \right) \right. \\ \left. + \, C_{\varphi } \left( L_{\varphi \varphi , t,\text{ norm }}^2 + C_t \, L_{\varphi \varphi ,tt,\text{ norm }}^2 \right) \right) /(\sigma _{\varphi }^{2\varGamma _{\varphi }} \sigma _{t}^{2 \varGamma _t}), \end{aligned}$$

where the individual components in this expression are defined from space-time separable receptive fields according to

19 $$\begin{aligned} L_{\varphi ,t,\text{ norm }}(x_1, x_2, t;\; \sigma _{\varphi }, \sigma _t, 0, \varSigma _{\varphi }) =\\ = T_{\varphi ,t,\text{ norm }}(x_1, x_2, t;\; \sigma _{\varphi }, \sigma _t, 0, \varSigma _{\varphi }) * f(x_1, x_2, t), \end{aligned}$$

20 $$\begin{aligned} L_{\varphi ,tt,\text{ norm }}(x_1, x_2, t;\; \sigma _{\varphi }, \sigma _t, 0, \varSigma _{\varphi }) =\\ = T_{\varphi ,tt,\text{ norm }}(x_1, x_2, t;\; \sigma _{\varphi }, \sigma _t, 0, \varSigma _{\varphi }) * f(x_1, x_2, t), \end{aligned}$$

21 $$\begin{aligned} L_{\varphi \varphi ,t,\text{ norm }}(x_1, x_2, t;\; \sigma _{\varphi }, \sigma _t, 0, \varSigma _{\varphi }) =\\ = T_{\varphi \varphi ,t,\text{ norm }}(x_1, x_2, t;\; \sigma _{\varphi }, \sigma _t, 0, \varSigma _{\varphi }) * f(x_1, x_2, t), \end{aligned}$$

22 $$\begin{aligned} L_{\varphi \varphi ,tt,\text{ norm }}(x_1, x_2, t;\; \sigma _{\varphi }, \sigma _t, 0, \varSigma _{\varphi }) =\\ = T_{\varphi \varphi ,tt,\text{ norm }}(x_1, x_2, t;\; \sigma _{\varphi }, \sigma _t, 0, \varSigma _{\varphi }) * f(x_1, x_2, t), \end{aligned}$$

with the underlying space-time separable spatio-temporal receptive fields $T_{\varphi ^m, t^n,\text{ norm }}(x_1, x_2, t;\; \sigma _{\varphi }, \sigma _t, 0, \varSigma _{\varphi })$ according to Eq. (13) for $v = 0$.

In the expression Eq. (18), the quasi-quadrature measure operates on both pairs of first- and second-order directional derivates as well as pairs of first- and second-order velocity-adapted derivatives simultaneously, aimed at balancing the responses of odd *vs.* even filter responses over both space or time in parallel.

This (new) spatio-temporal quasi-quadrature measure constitutes an extension of the spatio-temporal quasi-quadra-ture measure in (Lindeberg 2018 Section 4.2), by being additionally adapted to be selective to particular image orientations over the spatial domain, as opposed to being rotationally isotropic, as well as being based on affine Gaussian derivative operators, as opposed to partial derivatives of rotationally symmetric Gaussian kernels over the spatial domain.

This oriented spatio-temporal quasi-quadrature measure will specifically inherit the qualitative properties of the previously presented purely spatial oriented quasi-quadrature measure Eq. (40), in that it will be polarity-independent as well as much less sensitive to the phase in the input video data compared to the above models of simple cells.

#### Model based on velocity-adapted receptive fields

If we would compute the above spatio-temporal quasi-quadrature measure based on velocity-adapted spatio-tempo-ral receptive fields, then the quasi-quadrature measure would be zero if the velocity vector of the velocity-adapted receptive fields is equal to the velocity-value of the moving sine wave pattern. To define a quasi-quadrature measure that instead will give a maximally strong response if the velocity vector is adapted to the velocity vector of a moving pattern, we do therefore instead define a quasi-quadrature measure for velocity-adapted receptive fields by extending the purely spatial quasi-quadrature measure Eq. (10) to operate on spatial receptive fields complemented by a temporal smoothing stage, *i.e.*, velocity-adapted receptive fields for zero order of temporal differentiation ($n = 0$ in Eq. (13)):

23 $$\begin{aligned} (\mathcal{Q}_{\varphi ,\text{ vel },\text{ norm }} L) = \sqrt{\frac{L_{\varphi ,\text{ norm }}^2 + \, C_{\varphi } \, L_{\varphi \varphi ,\text{ norm }}^2}{\sigma _{\varphi }^{2\varGamma }}}, \end{aligned}$$

where the individual components in this expression are defined from space-time separable receptive fields according to

24 $$\begin{aligned} L_{\varphi ,\text{ norm }}(x_1, x_2, t;\; \sigma _{\varphi }, \sigma _t, v, \varSigma _{\varphi }) =\\ = T_{\varphi ,\text{ norm }}(x_1, x_2, t;\; \sigma _{\varphi }, \sigma _t, v, \varSigma _{\varphi }) * f(x_1, x_2, t), \end{aligned}$$

25 $$\begin{aligned} L_{\varphi \varphi ,\text{ norm }}(x_1, x_2, t;\; \sigma _{\varphi }, \sigma _t, v, \varSigma _{\varphi }) =\\ = T_{\varphi \varphi ,\text{ norm }}(x_1, x_2, t;\; \sigma _{\varphi }, \sigma _t, v, \varSigma _{\varphi }) * f(x_1, x_2, t), \end{aligned}$$

with the underlying space-time separable spatio-temporal receptive fields $T_{\varphi ^m, t^n,\text{ norm }}(x_1, x_2, t;\; \sigma _{\varphi }, \sigma _t, v, \varSigma _{\varphi })$ according to Eq. (13) for $n = 0$.

Again, the intention behind this (also new) definition is that also this spatio-temporal quasi-quadrature measure should be both polarity-independent with respect to the input and much less sensitive to the phase in the input compared to the velocity-adapted spatio-temporal models of simple cells, while also in conceptual agreement with the energy models of complex cells.

## Orientation selectivity properties for models of simple cells and complex cells in terms of affine Gaussian derivatives

In this section, we will theoretically analyze the orientation selectivity properties of the above described purely spatial as well as joint spatio-temporal models for receptive fields, when exposed to sine wave patterns with different orientations in relation to the preferred orientation of the receptive field.

### Motivation for separate analyses for the different classes of purely spatial or joint spatio-temporal receptive field models, as well as motivation for studying the orientation selectivity curves for models of both simple cells and complex cells

A main reason for performing separate analyses for both purely spatial and joint spatio-temporal models, is that, *a priori*, it may not be clear how the results from an analysis of the orientation selectivity of a purely spatial model would relate to the results from an orientation selectivity analysis of a joint spatio-temporal model. Furthermore, within the domain of joint spatio-temporal models, it is not *a priori* clear how the results from an orientation selectivity analysis of a space-time separable spatio-temporal model would relate to the results for a velocity-adapted spatio-temporal model. For this reason, we will perform separate individual analyses for these three main classes of either purely spatial or joint spatio-temporal receptive field models.

Additionally, it may furthermore not be *a priori* clear how the results from orientation selectivity analyses of models of simple cells that have different numbers of dominant spatial or spatio-temporal lobes would relate to each other, nor how the results from an orientation selectivity analysis of a model of a complex cell would relate to the analysis of orientation selectivity of models of simple cells. For this reason, we will also perform separate analyses for models of simple and complex cells, for each one of the above three main classes of receptive field models, which leads to a total number of $(2 + 1) + (2 \times 2 + 1) + (2 + 1) = 11$ separate analyses, in the cases of either purely spatial, space-time separable or velocity-adapted receptive spatio-temporal fields, respectively, given that we restrict ourselves to simple cells that can be modelled in terms of either first- or second-order spatial and/or temporal derivatives of affine Gaussian kernels. The reason why there are $2 \times 2 + 1 = 5$ subcases for the space-time separable spatio-temporal model is that we consider all the possible combinations of first- and second-order spatial derivatives with all possible combinations of first- and second-order temporal derivatives.

As will be demonstrated from the results, to be summarized in Table 1, the results from the orientation selectivity of the simple cells will turn out be similar within the class of purely first-order simple cells between the three classes of receptive field models (purely spatial, space-time separable or velocity-adapted spatio-temporal), as well as also similar within the class of purely second-order simple cells between the three classes of receptive field models. Regarding the models of complex cells, the results of the orientation selectivity analysis will be similar between the purely spatial and the velocity-adapted spatio-temporal receptive field models, whereas the results from the space-time separable spatio-temporal model of complex cells will differ from the other two. The resulting orientation selectivity curves will, however, differ between the sets of (i) first-order simple cells, (ii) second-order simple cells and (iii) complex cells.

The resulting special handling of the different cases that will arise from this analysis, with their different response characteristics, is particularly important, if one wants to fit parameterized models of orientation selectivity curves to actual neurophysiological data. If we want to relate neurophysiological findings measured for neurons, that are exposed to time-varying visual stimuli, or for neurons that have a strong dependency on temporal variations in the visual stimuli, then our motivation for performing a genuine spatio-temporal analysis for each one of the main classes of spatio-temporal models for simple cells, is to reduce the explanatory gap between the models and actual biological neurons.

**Table 1:** Summary of the forms of the orientation selectivity functions derived from the theoretical models of simple cells and complex cells based on the generalized Gaussian derivative model for visual receptive fields, in the cases of either (i) purely spatial models, (ii) space-time separable spatio-temporal models and (iii) velocity-adapted spatio-temporal models

	Purely spatial model	Space-time separable spatio-temporal model	Velocity-adapted spatio-temporal model
First-order simple cell	$\frac{\left| \cos \theta \right| }{\sqrt{\cos ^2 \theta + \kappa ^2 \sin ^2\theta }}$	$\frac{\left| \cos \theta \right| }{\sqrt{\cos ^2 \theta + \kappa ^2 \sin ^2\theta }}$	$\frac{\left| \cos \theta \right| }{\sqrt{\cos ^2 \theta + \kappa ^2 \sin ^2\theta }}$
Second-order simple cell	$\frac{\cos ^2 \theta }{\cos ^2 \theta + \kappa ^2 \sin ^2\theta }$	$\frac{\cos ^2 \theta }{\cos ^2 \theta + \kappa ^2 \sin ^2\theta }$	$\frac{\cos ^2 \theta }{\cos ^2 \theta + \kappa ^2 \sin ^2\theta }$
Complex cell	$\frac{\left| \cos \theta \right| ^{3/2}}{\left( \cos ^2 \theta + \kappa ^2 \sin ^2\theta \right) ^{3/4}}$	$\frac{\left| \cos \theta \right| \, \sqrt{2 + \kappa ^2 + (2 - \kappa ^2) \cos 2 \theta }}{\cos ^2\theta + \kappa ^2 \sin ^2\theta }$	$\frac{\left| \cos \theta \right| ^{3/2}}{\left( \cos ^2 \theta + \kappa ^2 \sin ^2\theta \right) ^{3/4}}$

Concerning the notation, the term “first-order simple cell” means a model of a simple cell that corresponds to a first-order directional derivative of an affine Gaussian kernel over the spatial domain, whereas the term “second-order simple cell” means a model of a simple cell that corresponds to a second-order directional derivative of an affine Gaussian kernel over the spatial domain. As we can see from the table, the form of the orientation selectivity function is similar for all the models of first-order simple cells. The form of the orientation selectivity function is also similar for all the models of second-order simple cells. For complex cells, the orientation selectivity function of the space-time separable model is, however, different from the orientation selectivity function of the purely spatial model and the velocity-adapted spatio-temporal model, which both have similar orientation selectivity functions. Note, in particular, that common for all these models is the fact that the degree of orientation selectivity increases with the scale parameter ratio $\kappa = \sigma _2/\sigma _1$, which is the ratio between the scale parameter $\sigma _2$ in the direction $\bot \varphi$ perpendicular to the preferred orientation $\varphi$ of the receptive field and the scale parameter $\sigma _1$ in the preferred orientation $\varphi$ of the receptive field. The shapes of the theoretically derived orientation selectivity curves do, however, notably differ between the classes of (i) first-order simple cells, (ii) second-order simple cells and (iii) complex cells

### Modelling scenario

**Fig. 1:**
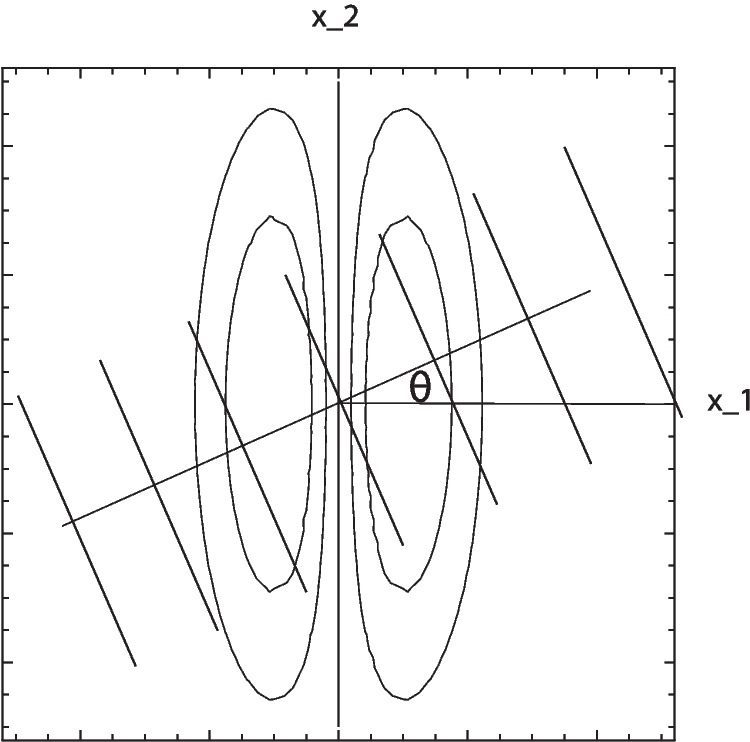
Schematic illustration of the modelling situation studied in the theoretical analysis, where the coordinate system is aligned to the preferred orientation $\varphi = 0$ of the receptive field, and the receptive field is then exposed to a sine wave pattern with inclination angle $\theta$. In this figure, the sine wave pattern is schematically illustrated by a set of level lines, overlayed onto a few level curves of a first-order affine Gaussian derivative kernel. (Horizontal axis: spatial coordinate $x_1$. Vertical axis: spatial coordinate $x_2$)

**Fig. 2:**
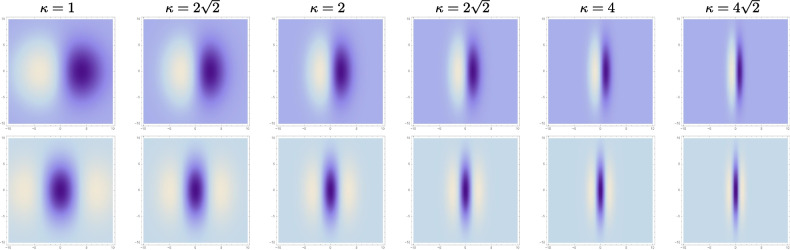
Affine Gaussian derivative receptive fields of orders 1 and 2 for the image orientation $\varphi = 0$, with the scale parameter ratio $\kappa = \sigma _2/\sigma _1$ increasing from 1 to $4\sqrt{2}$ according to a logarithmic distribution, from left to right, with the larger of these scale parameters $\sigma _2$ kept constant. (top row) First-order directional derivatives of affine Gaussian kernels according to Eq. (1) for $m = 1$. (bottom row) Second-order directional derivatives of affine Gaussian kernels according to Eq. (1) for $m = 2$. (Horizontal axes: image coordinate $x_1$. Vertical axes: image coordinate $x_2$)

For simplicity of analysis, and without loss of generality, we will henceforth align the coordinate system with the preferred orientation of the receptive field, in other words choosing the coordinate system such that the orientation angle $\varphi = 0$. Then, we will expose this receptive field to static or moving sine wave patterns, that are oriented with respect to an inclination angle $\theta$ relative to the resulting horizontal $x_1$-direction, as illustrated in Fig. 1. Figure 2 shows maps of the underlying first-order and second-order Gaussian derivatives for different degrees of elongation of the receptive fields.

By necessity, the presentation that will follow will be somewhat technical, since we will analyze the properties of our mathematical models for the receptive fields of simple and complex cells for three different main cases of either (i) purely spatial receptive fields, (ii) space-time separable spatio-temporal receptive fields and (iii) velocity-adapted spatio-temporal receptive fields.

Readers, who may be more interested in the final results only and their biological implications, while not in the details of the mathematical modelling with its associated theoretical analysis, can without major loss of continuity proceed directly to Sect. 4.6, where a condensed overview is given of the derived orientation selectivity results.

Readers, who additionally is interested in getting just a brief overview of how the theoretical analysis is carried out, and the assumptions regarding the probing of the receptive fields that it rests upon, would be recommended to additionally read at least one of the theoretical modelling cases, where the purely spatial analysis in the following Sect. 4.3 then constitutes the simplest case.

**Fig. 3:**
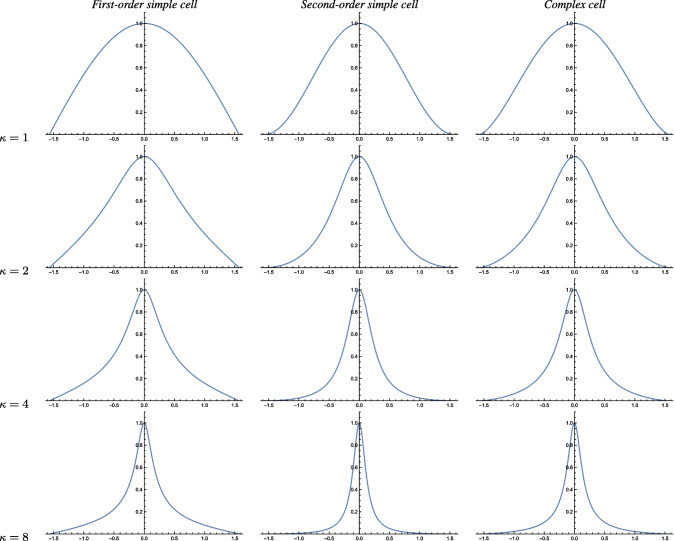
Graphs of the orientation selectivity for *purely spatial models* of (left column) simple cells in terms of first-order directional derivatives of affine Gaussian kernels, (middle column) simple cells in terms of second-order directional derivatives of affine Gaussian kernels and (right column) complex cells in terms of directional quasi-quadrature measures that combine the first- and second-order simple cell responses in a Euclidean way for $C = 1/\sqrt{2}$, and shown for different values of the ratio $\kappa$ between the spatial scale parameters in the vertical *vs.* the horizontal directions. Observe how the degree of orientation selectivity varies strongly depending on the eccentricity $\epsilon = 1/\kappa$ of the receptive fields. (top row) Results for $\kappa = 1$. (second row) Results for $\kappa = 2$. (third row) Results for $\kappa = 4$. (bottom row) Results for $\kappa = 8$. (Horizontal axes: orientation $\theta \in [-\pi /2, \pi /2]$. Vertical axes: Amplitude of the receptive field response relative to the maximum response obtained for $\theta = 0$)

### Analysis for purely spatial models of receptive fields

For the forthcoming purely spatial analysis, we will analyze the response properties of our spatial models of simple and complex cells to sine wave patterns with angular frequency $\omega$ and orientation $\theta$ of the form

27 $$\begin{aligned} f(x_1, x_2) = \sin \left( \omega \cos (\theta ) \, x_1 + \omega \sin (\theta ) \, x_2+ \beta \right) . \end{aligned}$$

#### First-order simple cell

Consider a simple cell that is modelled as a first-order scale-normalized derivative of an affine Gaussian kernel (according to Eq. (1) for $m = 1$), and oriented in the horizontal $x_1$-direction (for $\varphi = 0$) with spatial scale parameter $\sigma _1$ in the horizontal $x_1$-direction and spatial scale parameter $\sigma _2$ in the vertical $x_2$-direction, and thus a spatial covariance matrix of the form $\varSigma _0 = \operatorname {diag}(\sigma _1^2, \sigma _2^2)$:

28 $$\begin{aligned} {\begin{matrix}&T_{0,\text{ norm }}(x_1, x_2;\; \sigma _1, \sigma _2) = \end{matrix}}\nonumber \\ {\begin{matrix}&= \frac{\sigma _1}{2 \pi \sigma _1 \sigma _2} \, \partial _{x_1} \left( e^{-x_1^2/2\sigma _1^2 - x_2^2/2\sigma _2^2} \right) \end{matrix}}\nonumber \\ {\begin{matrix}&= - \frac{x_1}{2 \pi \sigma _1^2 \sigma _2} \, e^{-x_1^2/2\sigma _1^2 - x_2^2/2\sigma _2^2}. \end{matrix}} \end{aligned}$$

The corresponding receptive field response is then, after solving the convolution integral^6^ in Mathematica,

29 $$\begin{aligned} {\begin{matrix} L_{0,\text{ norm }}(x_1, x_2;\; \sigma _1, \sigma _2) = \end{matrix}}\nonumber \\ {\begin{matrix}&= \int _{\xi _1 = -\infty }^{\infty } \int _{\xi _2 = -\infty }^{\infty } T_{0,\text{ norm }}(\xi _1, \xi _2;\; \sigma _1, \sigma _2) \end{matrix}}\nonumber \\ {\begin{matrix}&{\hspace{85.0pt}} \times f(x_1 - \xi _1, x_2 - \xi _2) \, d \xi _1 \xi _2 \end{matrix}}\nonumber \\ {\begin{matrix}&= \omega \, \sigma _1 \cos (\theta ) \, e^{-\frac{1}{2} \omega ^2 (\sigma _1^2 \cos ^2 \theta + \sigma _2^2 \sin ^2 \theta )} \end{matrix}}\nonumber \\ {\begin{matrix}&{\hspace{17.0pt}} \times \cos ( \omega \cos (\theta ) \, x_1 + \omega \sin (\theta ) \, x_2+ \beta ), \end{matrix}} \end{aligned}$$

*i.e.*, a cosine wave with amplitude

30 $$\begin{aligned} A_{\varphi }(\theta , \omega ;\; \sigma _1, \sigma _2) = \omega \, \sigma _1 \left| \cos \theta \right| \, e^{-\frac{1}{2} \omega ^2 (\sigma _1^2 \cos ^2 \theta + \sigma _2^2 \sin ^2 \theta )}. \end{aligned}$$

If we assume that the receptive field is fixed, then the amplitude of the response will depend strongly on the angular frequency $\omega$ of the sine wave. The value first increases because of the factor $\omega$ and then decreases because of the exponential decrease with $\omega ^2$.

If we assume that a biological experiment regarding orientation selectivity is carried out in such a way that the angular frequency is varied for each inclination angle $\theta$, and then that the result is for each value of $\theta$ reported for the angular frequency $\hat{\omega }$ that leads to the maximum response, then we can determine this value of $\hat{\omega }$ by differentiating $A_{\varphi }(\theta , \omega ;\; \sigma _1, \sigma _2)$ with respect to $\omega$ and setting the derivative to zero, which gives:

31 $$\begin{aligned} \hat{\omega }_{\varphi } = \frac{1}{\sqrt{\sigma _1^2 \cos ^2 \theta + \sigma _2^2 \sin ^2 \theta }}. \end{aligned}$$

Inserting this value into $A_{\varphi }(\theta , \omega ;\; \sigma _1, \sigma _2)$, and introducing a scale parameter ratio $\kappa$ such that

32 $$\begin{aligned} \sigma _2 = \kappa \, \sigma _1, \end{aligned}$$

which implies

33 $$\begin{aligned} \hat{\omega }_{\varphi } = \frac{1}{\sigma _1 \sqrt{\cos ^2 \theta + \kappa ^2 \sin ^2 \theta }}, \end{aligned}$$

then gives the following orientation selectivity measure

34 $$\begin{aligned} A_{\varphi ,\max }(\theta , \; \kappa ) = \frac{\left| \cos \theta \right| }{\sqrt{e} \sqrt{\cos ^2 \theta + \kappa ^2 \sin ^2\theta }}. \end{aligned}$$

Note, specifically, that this amplitude measure is independent of the spatial scale parameter $\sigma _1$ of the receptive field, which, in turn, is a consequence of the scale-invariant nature of differential expressions in terms of scale-normalized derivatives for scale normalization parameter $\gamma = 1$.

The left column in Fig. 3 shows the result of plotting the measure $A_{\varphi ,\max }(\theta ;\; \kappa )$ of the orientation selectivity as function of the inclination angle $\theta$ for a few values of the scale parameter ratio $\kappa$, with the values rescaled such that the peak value for each graph is equal to 1. As can be seen from the graphs, the degree of orientation selectivity increases strongly with the value of the spatial scale ratio parameter $\kappa$.

#### Second-order simple cell

Consider next a simple cell that can be modelled as a second-order scale-normalized derivative of an affine Gaussian kernel (according to Eq. (1) for $m = 2$), and oriented in the horizontal $x_1$-direction (for $\varphi = 0$) with spatial scale parameter $\sigma _1$ in the horizontal $x_1$-direction and spatial scale parameter $\sigma _2$ in the vertical $x_2$-direction, and thus again with a spatial covariance matrix of the form $\varSigma _0 = \operatorname {diag}(\sigma _1^2, \sigma _2^2)$:

35 $$\begin{aligned} {\begin{matrix}&T_{00,\text{ norm }}(x_1, x_2;\; \sigma _1, \sigma _2) = \end{matrix}}\nonumber \\ {\begin{matrix}&= \frac{\sigma _1^2}{2 \pi \sigma _1 \sigma _2} \, \partial _{x_1 x_1} \left( e^{-x_1^2/2\sigma _1^2 - x_2^2/2 \sigma _2^2} \right) \end{matrix}}\nonumber \\ {\begin{matrix}&= \frac{(x_1^2 - \sigma _1^2)}{2 \pi \sigma _1^3 \sigma _2} \, e^{-x_1^2/2\sigma _1^2 - x_2^2/2 \sigma _2^2}. \end{matrix}} \end{aligned}$$

The corresponding receptive field response is then, again after solving the convolution integral in Mathematica,

36 $$\begin{aligned} {\begin{matrix} L_{00,\text{ norm }}(x_1, x_2;\; \sigma _1, \sigma _2) = \end{matrix}}\nonumber \\ {\begin{matrix}&= \int _{\xi _1 = -\infty }^{\infty } \int _{\xi _2 = -\infty }^{\infty } T_{00,\text{ norm }}(\xi _1, \xi _2;\; \sigma _1, \sigma _2) \end{matrix}}\nonumber \\ {\begin{matrix}&{\hspace{85.0pt}} \times f(x_1 - \xi _1, x_2 - \xi _2) \, d \xi _1 \xi _2 \end{matrix}}\nonumber \\ {\begin{matrix}&= - \omega ^2 \, \sigma _1^2 \cos ^2 (\theta ) \, e^{-\frac{1}{2} \omega ^2 (\sigma _1^2 \cos ^2 \theta + \sigma _2^2 \sin ^2 \theta )} \end{matrix}}\nonumber \\ {\begin{matrix}&{\hspace{15.0pt}} \times \sin ( \omega \cos (\theta ) \, x_1 + \omega \sin (\theta ) \, x_2 + \beta ), \end{matrix}} \end{aligned}$$

*i.e.*, a sine wave with amplitude

37 $$\begin{aligned}&A_{\varphi \varphi }(\theta , \omega ;\; \sigma _1, \sigma _2)\nonumber \\&\quad = \omega ^2 \, \sigma _1^2 \cos ^2 (\theta ) \, e^{-\frac{1}{2} \omega ^2 (\sigma _1^2 \cos ^2 \theta + \sigma _2^2 \sin ^2 \theta )}. \end{aligned}$$

**Fig. 4:**
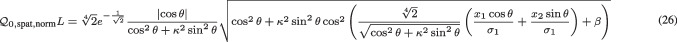
The expression for the oriented spatial quasi-quadrature measure $\mathcal{Q}_{0,\text{ spat },\text{ norm }} L$ in the purely spatial model Eq. (40) of a complex cell, when applied to a sine wave pattern of the form Eq. (27), for $\omega = \omega _\mathcal{Q}$ according to Eq. (41)

Again, also this expression first increases and then increases with the angular frequency $\omega$. Selecting again the value of $\hat{\omega }$ at which the amplitude of the receptive field response assumes its maximum over $\omega$ gives

38 $$\begin{aligned} \hat{\omega }_{\varphi \varphi } = \frac{\sqrt{2}}{\sigma _1 \sqrt{\cos ^2 \theta + \kappa ^2 \sin ^2 \theta }}, \end{aligned}$$

and implies that the maximum amplitude over spatial scales as function of the inclination angle $\theta$ and the scale parameter ratio $\kappa$ can be written

39 $$\begin{aligned} A_{\varphi \varphi ,\max }(\theta ;\; \kappa ) = \frac{2 \cos ^2 \theta }{e \left( \cos ^2 \theta + \kappa ^2 \sin ^2\theta \right) }. \end{aligned}$$

Again, this amplitude measure is also independent of the spatial scale parameter $\sigma _1$ of the receptive field, because of the scale-invariant property of scale-normalized derivatives, when the scale normalization parameter $\gamma$ is chosen as $\gamma = 1$.

The middle column in Fig. 3 shows the result of plotting the measure $A_{\varphi \varphi ,\max }(\theta ;\; \kappa )$ of the orientation selectivity as function of the inclination angle $\theta$ for a few values of the scale parameter ratio $\kappa$, with the values rescaled such that the peak value for each graph is equal to 1. Again, the degree of orientation selectivity increases strongly with the value of $\kappa$, as for the first-order model of a simple cell.

#### Complex cell

To model the spatial response of a complex cell according to the spatial quasi-quadrature measure in Eq. (10), we combine the responses of the first- and second-order simple cells for $\varGamma = 0$:

40 $$\begin{aligned} \mathcal{Q}_{0,\text{ spat },\text{ norm }} L = \sqrt{L_{0,\text{ norm }}^2 + C_{\varphi } \, L_{00,\text{ norm }}^2}, \end{aligned}$$

with $L_{0,\text{ norm }}$ according to Eq. (29) and $L_{00,\text{ norm }}$ according to Eq. (36). Choosing the angular frequency $\hat{\omega }$ as the geometric average of the angular frequencies for which the first- and second-order components of this entity assume their maxima over angular frequencies, respectively,

41 $$\begin{aligned} \hat{\omega }_\mathcal{Q} = \sqrt{\hat{\omega }_{\varphi } \, \hat{\omega }_{\varphi \varphi }} = \frac{\root 4 \of {2}}{\sigma _1 \sqrt{\cos ^2 \theta + \kappa ^2 \sin ^2 \theta }}, \end{aligned}$$

with $\hat{\omega }_{\varphi }$ according to Eq. (33) and $\hat{\omega }_{\varphi \varphi }$ according to Eq. (38). Again letting $\sigma _1 = \kappa \, \sigma _1$, and setting^7^ the relative weight between first- and second-order information to $C_{\varphi } = 1/\sqrt{2}$ according to Lindeberg (2018), gives the expression according to Eq. (26) in Fig. 4.

For inclination angle $\theta = 0$, that measure is spatially constant, in agreement with previous work on closely related isotropic purely spatial isotropic quasi-quadrature measures (Lindeberg, 2018). Then, the spatial phase dependency increases with increasing values of the inclination angle $\theta$. To select a single representative of those differing representations, let us choose the geometric average of the extreme values, which then assumes the form

42 $$\begin{aligned} A_{\mathcal{Q},\text{ spat }}(\theta ;\; \kappa ) = \frac{\root 4 \of {2} \, \left| \cos \theta \right| ^3}{\sqrt{e} \left( \cos ^2 \theta + \kappa ^2 \sin ^2\theta \right) ^{3/4}}. \end{aligned}$$

The right column in Fig. 3 shows the result of plotting the measure $A_{\mathcal{Q},\text{ spat }}(\theta ;\; \kappa )$ of the orientation selectivity as function of the inclination angle $\theta$ for a few values of the scale parameter ratio $\kappa$, with the values rescaled such that the peak value for each graph is equal to 1. As can be seen from the graphs, the degree of orientation selectivity increases strongly with the value of $\kappa$ also for this model of a complex cell, and in a qualitatively similar way as for the simple cell models.

### Analysis for space-time separable models of spatio-temporal receptive fields

To simplify the main flow through the paper, the detailed analysis of the orientation selectivity properties for the space-time separable models for the receptive fields of simple and complex cells is given in Appendix A.1, with the main results summarized in Sect. 4.6. Readers, who are mainly interested in understanding the principles by which the theoretical analysis is carried out, can proceed directly to Sect. 4.5, without major loss of continuity.

The main conceptual difference with the analysis of the space-time separable spatio-temporal receptive field models in Appendix A.1, compared to the purely spatial models in Sect. 4.3 or the velocity-adapted spatio-temporal receptive field models in Sect. 4.5, is that the space-time separable spatio-temporal receptive field models in Appendix A.1 do additionally involve derivatives with respect to time.

**Fig. 5:**
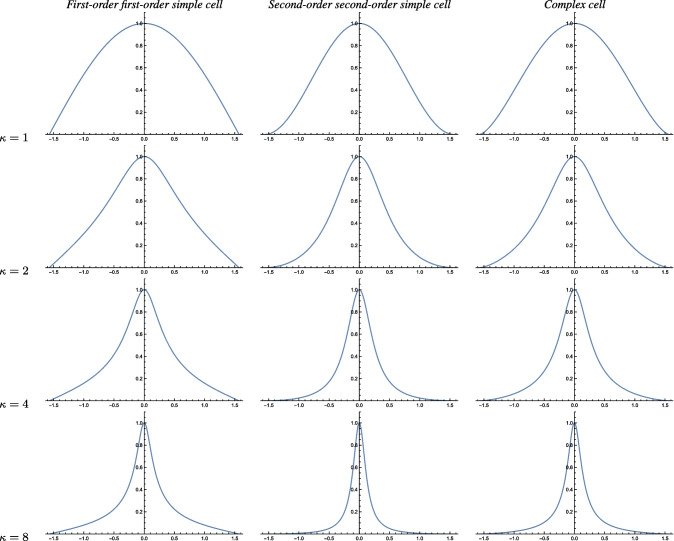
Graphs of the orientation selectivity for *velocity-adapted spatio-temporal models* of (left column) simple cells in terms of first-order directional derivatives of affine Gaussian kernels combined with zero-order temporal Gaussian kernels, (middle column) simple cells in terms of second-order directional derivatives of affine Gaussian kernels combined with zero-order temporal Gaussian kernels and (right column) complex cells in terms of directional quasi-quadrature measures that combine the first- and second-order simple cell responses in a Euclidean way for $C_{\varphi } = 1/\sqrt{2}$ shown for different values of the ratio $\kappa$ between the spatial scale parameters in the vertical *vs.* the horizontal directions. Observe how the degree of orientation selectivity varies strongly depending on the eccentricity $\epsilon = 1/\kappa$ of the receptive fields. (top row) Results for $\kappa = 1$. (second row) Results for $\kappa = 2$. (third row) Results for $\kappa = 4$. (bottom row) Results for $\kappa = 8$. (Horizontal axes: orientation $\theta \in [-\pi /2, \pi /2]$. Vertical axes: Amplitude of the receptive field response relative to the maximum response obtained for $\theta = 0$)

### Analysis for velocity-adapted spatio-temporal models of receptive fields

Let us next analyze the response properties of non-separable velocity-adapted spatio-temporal receptive fields to a moving sine wave of the form

44 $$\begin{aligned} f(x_1, x_2, t) = \\ = \sin \left( \omega \cos (\theta ) \, x_1 + \omega \sin (\theta ) \, x_2 - u \, t + \beta \right) . \end{aligned}$$

Based on the observation that the response properties of temporal derivatives will be zero, if the (scalar) velocity *v* of the spatio-temporal receptive field model is adapted to the (scalar) velocity *u* of the moving sine wave, we will study the case when the temporal order of differentiation *n* is zero.

#### First-order simple cell

Consider a velocity-adapted receptive field corresponding to a *first-order* scale-normalized Gaussian derivative with scale parameter $\sigma _1$ and velocity *v* in the horizontal $x_1$-direction, a zero-order Gaussian kernel with scale parameter $\sigma _2$ in the vertical $x_2$-direction, and a zero-order Gaussian derivative with scale parameter $\sigma _t$ in the temporal direction, corresponding to $\varphi = 0$, $v = 0$, $\varSigma _0 = \operatorname {diag}(\sigma _1^2, \sigma _2^2)$, $m = 1$ and $n = 0$ in Eq. (13):

45 $$\begin{aligned} {\begin{matrix}&T_{0,\text{ norm }}(x_1, x_2, t;\; \sigma _1, \sigma _2, \sigma _t) = \end{matrix}}\nonumber \\ {\begin{matrix}&= \frac{\sigma _1}{(2 \pi )^{3/2} \, \sigma _1 \sigma _2 \sigma _t} \, \partial _{x_1} \left. \left( e^{-x_1^2/2\sigma _1^2 - x_2^2/2 \sigma _2^2 - t^2/2\sigma _t^2} \right) \right| _{x_1 \rightarrow x_1-v t} \end{matrix}}\nonumber \\ {\begin{matrix}&= \frac{(x_1 - v t)}{(2 \pi )^{3/2} \, \sigma _1^2 \sigma _2 \sigma _t} \, e^{-(x_1- vt)^2/2\sigma _1^2 - x_2^2/2 \sigma _2^2 - t^2/2\sigma _t^2}. \end{matrix}} \end{aligned}$$

The corresponding receptive field response is then, after solving the convolution integral in Mathematica,

46 $$\begin{aligned} {\begin{matrix} L_{0,\text{ norm }}(x_1, x_2, t;\; \sigma _1, \sigma _2, \sigma _t) = \end{matrix}}\nonumber \\ {\begin{matrix}&= \int _{\xi _1 = -\infty }^{\infty } \int _{\xi _2 = -\infty }^{\infty } \int _{\zeta = -\infty }^{\infty } T_{0,\text{ norm }}(\xi _1, \xi _2, \zeta ;\; \sigma _1, \sigma _2, \sigma _t) \end{matrix}}\nonumber \\ {\begin{matrix}&{\hspace{85.0pt}} \times f(x_1 - \xi _1, x_2 - \xi _2, t - \zeta ) \, d \xi _1 \xi _2 d\zeta \end{matrix}}\nonumber \\ {\begin{matrix}&= \omega \, \sigma _1 \cos \theta \, \end{matrix}}\nonumber \\ {\begin{matrix}&{\hspace{15.0pt}} \times e^{-\frac{\omega ^2}{2} \left( \left( \sigma _1^2+\sigma _t^2 v^2\right) \cos ^2 (\theta ) +\sigma _2^2 \sin ^2 \theta -2 \sigma _t^2 u v \cos \theta +\sigma _t^2 u^2\right) } \end{matrix}}\nonumber \\ {\begin{matrix}&{\hspace{15.0pt}} \times \cos \left( \cos (\theta ) \, x_1 + \sin (\theta ) \, x_2 -\omega \, u \, t + \beta \right) , \end{matrix}} \end{aligned}$$

*i.e.*, a cosine wave with amplitude

47 $$\begin{aligned} {\begin{matrix}&A_{\varphi }(\theta , u, \omega ;\; \sigma _1, \sigma _2, \sigma _t) = \end{matrix}}\nonumber \\ {\begin{matrix}&= \omega \, \sigma _1 \left| \cos \theta \right| \, \end{matrix}}\nonumber \\ {\begin{matrix}&{\hspace{15.0pt}} \times e^{-\frac{\omega ^2}{2} \left( \cos ^2 \theta \left( \sigma _1^2+\sigma _t^2 v^2\right) +\sigma _2^2 \sin ^2 \theta -2 \sigma _t^2 u v \cos \theta +\sigma _t^2 u^2\right) }. \end{matrix}} \end{aligned}$$

Assume that a biological experiment regarding the response properties of the receptive field is performed by varying both the angular frequency $\omega$ and the image velocity *u* to get the maximum value of the response over these parameters. Differentiating the amplitude $A_{\varphi }$ with respect to $\omega$ and *u* and setting these derivatives to zero then gives

48 $$\begin{aligned} \hat{\omega }_{\varphi } = \frac{1}{\sigma _1 \sqrt{\cos ^2 \theta + \kappa ^2 \sin ^2 \theta }}, \end{aligned}$$

49 $$\begin{aligned} \hat{u}_{\varphi } = v \cos \theta . \end{aligned}$$

Inserting these values into $A_{\varphi }(\theta , u, \omega ;\; \sigma _1, \sigma _2, \sigma _t)$ then gives the following orientation selectivity measure

50 $$\begin{aligned} A_{\varphi ,\max }(\theta , \; \kappa ) = \frac{\left| \cos \theta \right| }{\sqrt{e} \, \sqrt{\cos ^2 \theta + \kappa ^2 \sin ^2\theta }}. \end{aligned}$$

The left column in Fig. 5 shows the result of plotting the measure $A_{\varphi ,\max }(\theta ;\; \kappa )$ of the orientation selectivity as function of the inclination angle $\theta$ for a few values of the scale parameter ratio $\kappa$, with the values rescaled such that the peak value for each graph is equal to 1. As we can see from the graphs, as for the previous purely spatial models of the receptive fields, as well as for the previous space-time separable model of the receptive fields, the degree of orientation selectivity increases strongly with the value of $\kappa$.

#### Second-order simple cell

Consider next a velocity-adapted receptive field corresponding to a *second-order* scale-normalized Gaussian derivative with scale parameter $\sigma _1$ and velocity *v* in the horizontal $x_1$-direction, a zero-order Gaussian kernel with scale parameter $\sigma _2$ in the vertical $x_2$-direction, and a zero-order Gaussian derivative with scale parameter $\sigma _t$ in the temporal direction, corresponding to $\varphi = 0$, $v = 0$, $\varSigma _0 = \operatorname {diag}(\sigma _1^2, \sigma _2^2)$, $m = 2$ and $n = 0$ in Eq. (13):

51 $$\begin{aligned} {\begin{matrix}&T_{00,\text{ norm }}(x_1, x_2, t;\; \sigma _1, \sigma _2, \sigma _t) = \end{matrix}}\nonumber \\ {\begin{matrix}&= \frac{\sigma _1^2}{(2 \pi )^{3/2} \, \sigma _1 \sigma _2 \sigma _t} \, \partial _{x_1 x_1} \left. \left( e^{-x_1^2/2\sigma _1^2 - x_2^2/2 \sigma _2^2 - t^2/2\sigma _t^2} \right) \right| _{x_1 \rightarrow x_1-v t} \end{matrix}}\nonumber \\ {\begin{matrix}&= \frac{((x_1 - v t)^2 - \sigma _1^2)}{(2 \pi )^{3/2} \, \sigma _1^3 \sigma _2 \sigma _t} \, e^{-(x_1 - v t)^2/2\sigma _1^2 - x_2^2/2 \sigma _2^2 - t^2/2\sigma _t^2}. \end{matrix}} \end{aligned}$$

**Fig. 6:**
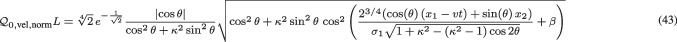
The expression for the oriented spatio-temporal quasi-quadrature measure $\mathcal{Q}_{0,\text{ vel },\text{ norm }} L$ in the velocity-adapted spatio-temporal model Eq. (23) of a complex cell, when applied to a sine wave pattern of the form Eq. (44), for $\omega = \omega _\mathcal{Q}$ according to Eq. (58) and $u = u_\mathcal{Q}$ according to Eq. (59)

The corresponding receptive field response is then, after solving the convolution integral in Mathematica,

52 $$\begin{aligned} {\begin{matrix} L_{00,\text{ norm }}(x_1, x_2, t;\; \sigma _1, \sigma _2, \sigma _t) = \end{matrix}}\nonumber \\ {\begin{matrix}&= \int _{\xi _1 = -\infty }^{\infty } \int _{\xi _2 = -\infty }^{\infty } \int _{\zeta = -\infty }^{\infty } T_{00,\text{ norm }}(\xi _1, \xi _2, \zeta ;\; \sigma _1, \sigma _2, \sigma _t) \end{matrix}}\nonumber \\ {\begin{matrix}&{\hspace{85.0pt}} \times f(x_1 - \xi _1, x_2 - \xi _2, t - \zeta ) \, d \xi _1 \xi _2 d\zeta \end{matrix}}\nonumber \\ {\begin{matrix}&= -\omega ^2 \sigma _1^2 \cos ^2 \theta \, \end{matrix}}\nonumber \\ {\begin{matrix}&{\hspace{15.0pt}} \times e^{-\frac{\omega ^2}{2} \left( \left( \sigma _1^2+\sigma _t^2 v^2\right) \cos ^2 \theta +\sigma _2^2 \sin ^2 \theta -2 \sigma _t^2 u v \cos \theta +\sigma _t^2 u^2\right) } \end{matrix}}\nonumber \\ {\begin{matrix}&{\hspace{15.0pt}} \times \cos \left( \sin (\theta ) \, x_1 + \sin (\theta ) \, x_2 - \omega \, u \, t + \beta \right) , \end{matrix}} \end{aligned}$$

*i.e.*, a sine wave with amplitude

53 $$\begin{aligned} {\begin{matrix} & A_{\varphi \varphi }(\theta , u, \omega ;\; \sigma _1, \sigma _2, \sigma _t) = \\ \end{matrix}}\nonumber \\ {\begin{matrix}&= \omega ^2 \sigma _1^2 \cos ^2 \theta \, \end{matrix}}\nonumber \\ {\begin{matrix}&{\hspace{15.0pt}} \times e^{-\frac{\omega ^2}{2} \left( \cos ^2 \theta \left( \sigma _1^2+\sigma _t^2 v^2\right) +\sigma _2^2 \sin ^2 \theta -2 \sigma _t^2 u v \cos \theta +\sigma _t^2 u^2\right) }. \end{matrix}} \end{aligned}$$

Assume that a biological experiment regarding the response properties of the receptive field is performed by varying both the angular frequency $\omega$ and the image velocity *u* to get the maximum value of the response over these parameters. Differentiating the amplitude $A_{\varphi \varphi }$ with respect to $\omega$ and *u* and setting these derivatives to zero then gives

54 $$\begin{aligned} \hat{\omega }_{\varphi \varphi } = \frac{\sqrt{2}}{\sigma _1 \sqrt{\cos ^2 \theta + \kappa ^2 \sin ^2 \theta }}, \end{aligned}$$

55 $$\begin{aligned} \hat{u}_{\varphi \varphi } = v \cos \theta . \end{aligned}$$

Inserting these values into $A_{\varphi \varphi }(\theta , u, \omega ;\; \sigma _1, \sigma _2, \sigma _t)$ then gives the following orientation selectivity measure

56 $$\begin{aligned} A_{\varphi \varphi ,\max }(\theta , \; \kappa ) = \frac{2 \cos ^2 \theta }{e \, (\cos ^2 \theta + \kappa ^2 \sin ^2\theta )}. \end{aligned}$$

The middle column in Fig. 5 shows the result of plotting the measure $A_{\varphi \varphi ,\max }(\theta ;\; \kappa )$ of the orientation selectivity as function of the inclination angle $\theta$ for a few values of the scale parameter ratio $\kappa$, with the values rescaled such that the peak value for each graph is equal to 1. Again, the degree of orientation selectivity increases strongly with the value of $\kappa$.

#### Complex cell

To model the spatial response of a complex cell according to the spatio-temporal quasi-quadrature measure Eq. (23) based on velocity-adapted spatio-temporal receptive fields, we combine the responses of the first- and second-order simple cells (for $\varGamma = 0$)

57 $$\begin{aligned} (\mathcal{Q}_{0,\text{ vel },\text{ norm }} L) = \sqrt{\frac{L_{0,\text{ norm }}^2 + \, C_{\varphi } \, L_{00,\text{ norm }}^2}{\sigma _{\varphi }^{2\varGamma }}}, \end{aligned}$$

with $L_{0,\text{ norm }}$ according to Eq. (46) and $L_{00,\text{ norm }}$ according to Eq. (52).

Selecting the angular frequency as the geometric average of the angular frequency values at which the above spatio-temporal simple cell models assume their maxima over angular frequencies, as well as using the same value of *u*,

58 $$\begin{aligned} \hat{\omega }_\mathcal{Q} = \sqrt{\hat{\omega }_{\varphi } \, \hat{\omega }_{\varphi \varphi }} = \frac{\root 4 \of {2}}{\sigma _1 \sqrt{\cos ^2 \theta + \kappa ^2 \sin ^2 \theta }}, \end{aligned}$$

with $\hat{\omega }_{\varphi }$ according to Eq. (48) and $\hat{\omega }_{\varphi \varphi }$ according to Eq. (54), as well as choosing the image velocity $\hat{u}$ as the same value as for which the above spatio-temporal simple cell models assume their maxima over the image velocity (Eqs. (49) and (55))

59 $$\begin{aligned} \hat{u}_\mathcal{Q} = v \cos \theta , \end{aligned}$$

as well as letting $\sigma _1 = \kappa \, \sigma _1$, and setting the relative weights between first- and second-order information to $C_{\varphi } = 1/\sqrt{2}$ and $C_t = 1/\sqrt{2}$ according to Lindeberg (2018), then gives the expression according to Eq. (43) in Fig. 6.

For inclination angle $\theta = 0$, that measure is spatially constant, in agreement with our previous purely spatial analysis, as well as in agreement with previous work on closely related isotropic spatio-temporal quasi-quadrature measures (Lindeberg, 2018). When the inclination angle increases, the phase dependency of the quasi-quadrature measure will, however, increase. To select a single representative of those differing representations, let us choose the geometric average of the extreme values, which then assumes the form

60 $$\begin{aligned} A_{\mathcal{Q},\text{ vel },\max }(\theta ;\; \kappa ) = \frac{\root 4 \of {2} \, \left| \cos \theta \right| ^{3/2} }{e^{1/\sqrt{2}} \, (\cos ^2 \theta + \kappa ^2 \sin ^2\theta )^{3/2}}. \end{aligned}$$

The right column in Fig. 5 shows the result of plotting the measure $A_{\mathcal{Q},\text{ vel },\max }(\theta ;\; \kappa )$ of the orientation selectivity as function of the inclination angle $\theta$ for a few values of the scale parameter ratio $\kappa$, with the values rescaled such that the peak value for each graph is equal to 1. Again, the degree of orientation selectivity increases strongly with the value of $\kappa$.

### Resulting models for orientation selectivity

Table 1 summarizes the results from the above theoretical analysis of the orientation selectivity for our idealized models of simple cells and complex cells, based on the generalized Gaussian derivative model for visual receptive fields, in the cases of either (i) purely spatial models, (ii) space-time separable spatio-temporal models and (iii) velocity-adapted spatio-temporal models. The overall methodology that we have used for deriving these results is by exposing each theoretical receptive field model to either purely spatial or joint spatio-temporal sine wave patterns, and measuring the response properties for different inclination angles $\theta$, at the angular frequency of the sine wave, as well as the image velocity of the spatio-temporal sine wave, at which these models assume their maximum response over variations of these probing parameters.

As can be seen from the table, the form of the orientation selectivity curve is similar for all the models of first-order simple cells, which correspond to first-order derivatives of affine Gaussian kernels over the spatial domain. The form of the orientation selectivity curve is also similar for all the models of second-order simple cells, which correspond to second-order derivatives of affine Gaussian kernels over the spatial domain. For complex cells, the form of the orientation selectivity curve for the space-time separable model is, however, different from the form of the orientation selectivity curve for the purely spatial model and the velocity-adapted spatio-temporal model, which both have a similar form for their orientation selectivity curves.

Note, in particular, that common for all these models is the fact that the degree of orientation selectivity increases with the scale parameter ratio $\kappa = \sigma _2/\sigma _1$, which is the ratio between the scale parameter $\sigma _2$ in the direction $\bot \varphi$ perpendicular to the preferred orientation $\varphi$ of the receptive field and the scale parameter $\sigma _1$ in the preferred orientation $\varphi$ of the receptive field. In other words, for higher values of $\kappa$, the form of the orientation selectivity curve is more narrow than the form of the orientation selectivity curve for a lower value of $\kappa$. The form of the orientation selectivity curve is also more narrow for a simple cell that can be modelled as a second-order directional derivative of an affine Gaussian kernel, than for a simple cell that can be modelled as a first order derivative of an affine Gaussian kernel.

In this respect, the theoretical analysis supports the conclusion that the degree of orientation selectivity of the receptive fields increases with the degree of anisotropy or elongation of the receptive fields, specifically the fact that highly anisotropic or elongated affine Gaussian derivative based receptive fields have higher degree of orientation selectivity than more isotropic affine Gaussian derivative based receptive fields.

The shapes of the resulting orientation selectivity curves do, however, notably differ between the classes of (i) first-order simple cells, (ii) second-order simple cells and (iii) complex cells. This property is important to take into account, if one aims at fitting parameterized models of orientation selectivity curves to neurophysiological measurements of corresponding data.

## Orientation selectivity analysis for an affine Gabor model of simple cells

In this section, we will perform an orientation selectivity analysis, as analytically similar as possible, for idealized receptive fields according to an affine Gabor model for purely spatial receptive fields.

Given two spatial scale parameters $\sigma _1$ and $\sigma _2$ and an angular frequency $\nu$, consider an affine Gabor model with a pair of purely spatial receptive fields of the form

61 $$\begin{aligned} {\begin{matrix} & T_{\text{ even }}(x_1, x_2;\; \sigma _1, \sigma _2, \nu )\\ & \quad = \frac{1}{2 \pi \sigma _1 \sigma _2} \, e^{- x_1^2/2 \sigma _1^2 - x_2^2/2 \sigma _2^2} \cos (\nu \, x_1), \end{matrix}}\end{aligned}$$

62 $$\begin{aligned} {\begin{matrix} & T_{\text{ odd }}(x_1, x_2;\; \sigma _1, \sigma _2, \nu )\\ & \quad = \frac{1}{2 \pi \sigma _1 \sigma _2} \, e^{- x_1^2/2 \sigma _1^2 - x_2^2/2 \sigma _2^2} \sin (\nu \, x_1). \end{matrix}} \end{aligned}$$

Let us, in analogy with the treatment in Sect. 4.3, subject these receptive fields to test probes of the form in Eq. (27)

63 $$\begin{aligned} f(x_1, x_2) = \sin \left( \omega \cos (\theta ) \, x_1 + \omega \sin (\theta ) \, x_2+ \beta \right) \end{aligned}$$

for different inclination angles $\theta$. The responses of the even and odd components of the Gabor pair to such test functions are given by

64 $$\begin{aligned} {\begin{matrix} L_{\text{ even }}(x_1, x_2;\; \sigma _1, \sigma _2, \nu ) = \end{matrix}}\nonumber \\ {\begin{matrix}&= \int _{\xi _1 = -\infty }^{\infty } \int _{\xi _2 = -\infty }^{\infty } T_{\text{ even }}(\xi _1, \xi _2;\; \sigma _1, \sigma _2, \nu ) \end{matrix}}\nonumber \\ {\begin{matrix}&{\hspace{85.0pt}} \times f(x_1 - \xi _1, x_2 - \xi _2) \, d \xi _1 \xi _2, \end{matrix}}\nonumber \\ {\begin{matrix} L_{\text{ odd }}(x_1, x_2;\; \sigma _1, \sigma _2, \nu ) = \end{matrix}}\nonumber \\ {\begin{matrix}&= \int _{\xi _1 = -\infty }^{\infty } \int _{\xi _2 = -\infty }^{\infty } T_{\text{ odd }}(\xi _1, \xi _2;\; \sigma _1, \sigma _2, \nu ) \end{matrix}}\nonumber \\ {\begin{matrix}&{\hspace{85.0pt}} \times f(x_1 - \xi _1, x_2 - \xi _2) \, d \xi _1 \xi _2. \end{matrix}} \end{aligned}$$

Solving these convolution integrals in Mathematica then gives

65 $$\begin{aligned} {\begin{matrix} L_{\text{ even }}(x_1, x_2;\; \sigma _1, \sigma _2, \nu ) = \end{matrix}}\nonumber \\ {\begin{matrix}&= \frac{1}{2} \left( e^{2 \nu \omega \sigma _1^2 \cos \theta }+1\right) \sin (\beta +\omega \, x_1 \cos \theta +\omega \, x_2 \sin \theta ) \times \end{matrix}}\nonumber \\ {\begin{matrix}&{\hspace{10.0pt}} \quad e^{\frac{1}{2} \left( -\nu ^2 \sigma _1^2-2 \nu \omega \sigma _1^2 \cos \theta -\omega ^2 \sigma _1^2 \cos ^2\theta -\omega ^2 \sigma _2^2 \sin ^2 \theta \right) }, \end{matrix}}\end{aligned}$$

66 $$\begin{aligned} {\begin{matrix} L_{\text{ odd }}(x_1, x_2;\; \sigma _1, \sigma _2, \nu ) = \end{matrix}}\nonumber \\ {\begin{matrix}&=\! -\frac{1}{2} \left( e^{2 \nu \omega \sigma _1^2 \cos \theta }-1\right) \cos (\beta +\omega \, x_1 \cos \theta +\omega \, x_2 \sin \theta ) \times \end{matrix}}\nonumber \\ {\begin{matrix}&{\hspace{10.0pt}} \quad e^{\frac{1}{2} \left( -\nu ^2 \sigma _1^2-2 \nu \omega \sigma _1^2 \cos \theta -\omega ^2 \sigma _1^2 \cos ^2\theta -\omega ^2 \sigma _2^2 \sin ^2 \theta \right) }. \end{matrix}} \end{aligned}$$

Unfortunately, it is hard to analytically determine the angular frequency $\omega$ of the test function that gives the strongest amplitude for the Gabor response obtained with a given frequency $\nu$. Therefore, we will in the following simply set $\omega = \nu$, implying that this analysis for the Gabor model will not be methodologically identical to the previous analysis for the affine Gaussian derivative model, in the sense that we will not optimize the angular frequency $\omega$ of the test probe for each inclination angle $\theta$.

Letting additionally $\sigma _2 = \kappa \, \sigma _1$ for $\kappa > 1$, we obtain that the amplitudes of the two Gabor responses can be written:

67 $$\begin{aligned} {\begin{matrix} A_{\text{ even }}(\theta ) =\! \frac{1}{2} \left( e^{2 \nu ^2 \sigma _1^2 \cos \theta }+1 \right) e^{- \nu ^2 \sigma _1^2 \cos ^2 \left( \frac{\theta }{2}\right) (\kappa ^2-1)(1 - \cos \theta )}, \end{matrix}}\end{aligned}$$

68 $$\begin{aligned} {\begin{matrix} A_{\text{ odd }}(\theta ) = \frac{1}{2} \left( e^{2 \nu ^2 \sigma _1^2 \cos \theta }-1 \right) e^{- \nu ^2 \sigma _1^2 \cos ^2 \left( \frac{\theta }{2}\right) (\kappa ^2-1)(1 - \cos \theta )}. \end{matrix}} \end{aligned}$$

In contrast to the previous analysis for affine Gaussian derivative models of simple cells, where we determined the angular frequency of the probing sine wave, to maximize the response given a value of the spatial scale parameter, and in this way eliminated the dependency of the orientation selectivity on the size of the spatial scale parameter in the idealized receptive field model, and in this way reduced the explicit orientation dependency to only depend on the ratio $\kappa$ between the scale parameters, we cannot, however, here eliminate the remaining parameters of the idealized Gabor model from the orientation selectivity analysis.

**Fig. 7:**
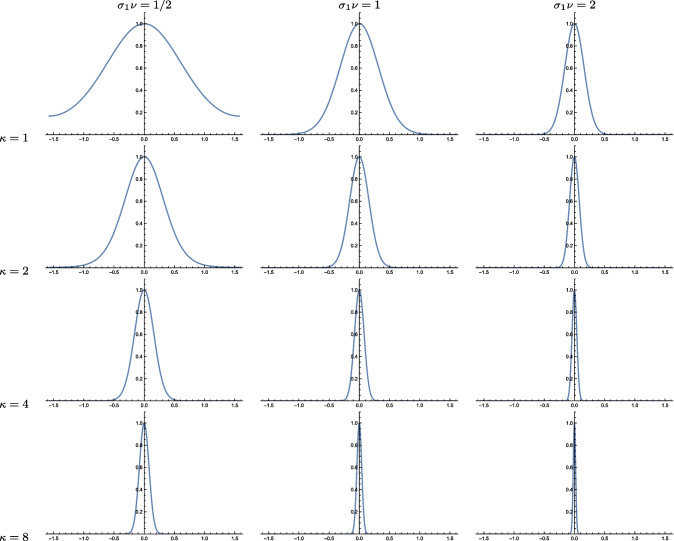
Graphs of the orientation selectivity of *purely spatial models* of a simple cell, using the *even component of the affine Gabor model* according to Eqs. (61) and (62). Observe how the degree of orientation selectivity depends on the scale parameter ratio $\kappa$, in a qualitatively similar manner as for the affine Gaussian derivative model for purely spatial receptive fields. For the affine Gabor model, the degree of orientation selectivity does, however, also depend strongly on the product of the two remaining parameters $\sigma _1 \, \nu$ in the model. (top row) Results for $\kappa = 1$. (second row) Results for $\kappa = 2$. (third row) Results for $\kappa = 4$. (bottom row) Results for $\kappa = 8$. (left column) Results for $\sigma _1 \nu = 1/2$. (middle column) Results for $\sigma _1 \nu = 1$. (right column) Results for $\sigma _1 \nu = 2$. (Horizontal axes: orientation $\theta \in [-\pi /2, \pi /2]$. Vertical axes: Amplitude of the receptive field response relative to the maximum response obtained for $\theta = 0$)

The affine Gabor model comprises four internal parameters for the receptive fields (the spatial scale parameters $\sigma _1$ and $\sigma _2$, the angular frequency $\nu$ as well as the here not explicitly modelled orientation angle $\varphi$), while the purely spatial affine Gaussian derivative model comprises only three internal parameters (the spatial scale parameters $\sigma _1$ and $\sigma _2$ as well as the in Sect. 4 neither explicitly modelled orientation angle $\varphi$).

The affine Gabor model, thus, comprises one more parameter than the affine Gaussian derivative model. The dependency on the remaining degrees of freedom (beyond the orientation $\varphi$), due to variations in $\sigma _1$ and $\nu$, is, however, of a special form, in the respect that the dependency only depends on the product between $\sigma _1$ and $\nu$. Therefore, we will have to, beyond a variability with respect to the scale parameter ratio $\kappa$, also investigate the behaviour with respect to variations of the product of the angular frequency $\nu$ and the remaining spatial scale parameter $\sigma _1$.

Figure 7 shows orientation selectivity curves for the even component of the affine Gabor pair for different combinations of the scale parameter ratio $\kappa = 1, 2, 4$ or 8, and the product $\sigma _1 \nu = 1/2, 1$ or 2. As can be seen from these graphs, the orientation selectivity becomes more narrow with increasing values of the scale parameter ratio $\kappa$, as for the affine Gaussian derivative model. The orientation selectivity does, however, also become more narrow when the product $\sigma _1 \, \nu$ increases.

Hence, from the conceptual background of whether one could possibly infer that the receptive fields ought to be more elongated, when the orientation selectivity becomes more narrow, one would not be able to make such a logical inference from a population of neurons according to the affine Gabor model, if the only *a priori* would be that the population would be generated from an expansion over all the four internal parameters $\sigma _1$, $\kappa$, $\nu$ and the orientation $\varphi$. If it, on the other hand, would be *a priori* known that the relationship between the angular frequency $\nu$ and the remaining spatial scale parameter $\sigma _1$ would be fixed to their product being held constant, then one would, in principle, be able to make such a logical inference.

To save space, we do not show the graphs for the corresponding orientation selectivity curves for the odd component of the Gabor pair, nor for the idealized model of a complex cell, obtained by squaring the responses from the odd and even components and then adding them, and finally taking the square root of the result:

69 $$\begin{aligned} {\begin{matrix} Q^2 = L_{\text{ even }}^2 + L_{\text{ odd }}^2 \end{matrix}}\nonumber \\ {\begin{matrix} = \frac{1}{4} e^{-\nu ^2 \sigma _1^2 \left( \kappa ^2 \sin ^2\theta +\cos ^2\theta +2 \cos \theta +1\right) } \times \end{matrix}}\nonumber \\ {\begin{matrix} {\hspace{10.0pt}} \left( 1 + e^{4 \nu ^2 \sigma _1^2 \cos \theta } \right. \end{matrix}}\nonumber \\ {\begin{matrix} {\hspace{10.0pt}} \left. \quad -2 \, e^{2 \nu ^2 \sigma _1^2 \cos \theta } \cos (2 (\beta +\nu \, x_1 \cos \theta +\nu \, x_2 \sin \theta )) \right) . \end{matrix}} \end{aligned}$$

The qualitative results, that receptive fields become more narrow as the scale parameter ratio $\kappa$ increases, or the product $\sigma _1 \nu$ of the two remaining parameters increases, are, however, similar. So are the results that the orientation selectivity of the receptive fields, however, also depends strongly upon the product $\sigma _1 \nu$.

We refrain from extending the analysis to possible spatio-temporal models, since it may not be fully clear how the spatio-temporal Gabor models should then be defined.

## Relations to compact quantitative measures of the degree of orientation selectivity

In Ringach et al. (2002), two compact measures of the orientation selectivity of biological receptive fields are used, in terms of either the circular variance (Mardia, 1972)

70 $$\begin{aligned} V = 1 - |R|, \end{aligned}$$

where the complex-valued resultant *R* is given by

71 $$\begin{aligned} R = \frac{\int _{\theta = - \pi }^{\pi } r(\theta ) \, e^{2 i \theta } d\theta }{\int _{\theta = - \pi }^{\pi } r(\theta ) \, d\theta }, \end{aligned}$$

or the orientation bandwidth *B*, defined as the value of *B* for which

72 $$\begin{aligned} \frac{r(B)}{r(0)} = \frac{1}{\sqrt{2}}. \end{aligned}$$

In the following, we will compute these compact measures for the orientation selectivity curves derived from the affine-Gaussian-derivative-based models of visual receptive fields, as summarized in Table 1.

**Fig. 8:**
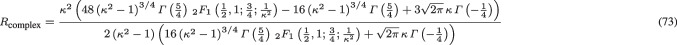
Explicit expression for the resultant *R* for (i) the purely spatial model of complex cells based on affine Gaussian derivatives, as well as for (ii) the velocity-adapted spatio-temporal model of complex cells based on affine Gaussian derivatives. The function $_2F_1(a, b; c; z)$ is the hypergeometric function $\operatorname {Hypergeometric2F1}[a, b, c, z]$ in Mathematica, while $\varGamma (z)$ denotes Euler’s Gamma function

### The resultants *R* of the orientation selectivity curves computed for the affine-Gaussian-derivative-based models of simple and complex cells

Due to the symmetry of the orientation selectivity curves in Table 1, the purely imaginary component of the resultant *R* will be zero for all the considered models of simple and complex cells in terms of affine-Gaussian-derivative-based receptive fields. In the following, we therefore focus solely on the purely real component of the resultants.

For the first-order simple cells, we obtain, after solving the resulting integrals in Mathematica:

77 $$\begin{aligned} {\begin{matrix} R_{\text{ simple },1}&= \frac{\int _{\theta = -\pi /2}^{\pi /2} \frac{ \cos \theta }{\sqrt{\cos ^2 \theta + \kappa ^2 \sin ^2\theta }} \, \cos 2 \theta \, d\theta }{\int _{\theta = -\pi /2}^{\pi /2} \frac{ \cos \theta }{\sqrt{\cos ^2 \theta + \kappa ^2 \sin ^2\theta }} \, d\theta } \end{matrix}}\nonumber \\ {\begin{matrix}&= \frac{\kappa \left( \kappa \cosh ^{-1} \kappa -\sqrt{\kappa ^2-1}\right) }{\left( \kappa ^2-1\right) \cosh ^{-1} \kappa }, \end{matrix}} \end{aligned}$$

and for the second-order simple cells:

78 $$\begin{aligned} {\begin{matrix} R_{\text{ simple },2}&= \frac{\int _{\theta = -\pi /2}^{\pi /2} \frac{\cos ^2 \theta }{\cos ^2 \theta + \kappa ^2 \sin ^2\theta } \, \cos 2 \theta \, d\theta }{\int _{\theta = -\pi /2}^{\pi /2} \frac{\cos ^2 \theta }{\cos ^2 \theta + \kappa ^2 \sin ^2\theta } \, d\theta } \end{matrix}}\nonumber \\ {\begin{matrix}&= \frac{\kappa }{\kappa +1}. \end{matrix}} \end{aligned}$$

For both the purely spatial model of complex cells based on affine Gaussian derivatives, as well as for the velocity-adapted model of complex cells based on affine Gaussian derivatives, we do in a similar manner get

79 $$\begin{aligned} {\begin{matrix} R_{\text{ complex }}&= \frac{\int _{\theta = -\pi /2}^{\pi /2} \frac{\cos ^{3/2} \theta }{\left( \cos ^2 \theta + \kappa ^2 \sin ^2\theta \right) ^{3/4}} \, \cos 2 \theta \, d\theta }{\int _{\theta = -\pi /2}^{\pi /2} \frac{\cos ^{3/2} \theta }{\left( \cos ^2 \theta + \kappa ^2 \sin ^2\theta \right) ^{3/4}} \, d\theta } \end{matrix}} \end{aligned}$$

with the explicit expression for that result in Fig. 8.

For the space-time separable model of complex cells, the corresponding formulation

80 $$\begin{aligned} {\begin{matrix} R_{\text{ complex }-\text{ sep }}&= \frac{\int _{\theta = -\pi /2}^{\pi /2} \frac{\cos \theta \, \sqrt{2 + \kappa ^2 + (2 - \kappa ^2) \cos 2 \theta }}{\cos ^2\theta + \kappa ^2 \sin ^2\theta } \, \cos 2 \theta \, d\theta }{\int _{\theta = -\pi /2}^{\pi /2} \frac{\cos \theta \, \sqrt{2 + \kappa ^2 + (2 - \kappa ^2) \cos 2 \theta }}{\cos ^2\theta + \kappa ^2 \sin ^2\theta } \, d\theta } \end{matrix}} \end{aligned}$$

can be handled in closed form in Mathematica, but does unfortunately lead to an explicit expression for the result that is too complex to be reported here.

**Fig. 9:**
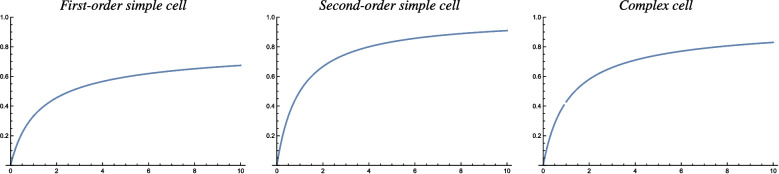
Graphs of the *resultant* *R* of the orientation selectivity curves as function of the scale parameter ratio $\kappa$ for the different models of visual receptive fields based on affine Gaussian derivatives. (left) First-order model of simple cell. (middle) Second-order model of simple cell. (right) Purely spatial model of complex cell or velocity-adapted model of complex cell. (Horizontal axes: scale parameter ratio $\kappa$. Vertical axes: resultant *R*)

**Fig. 10:**
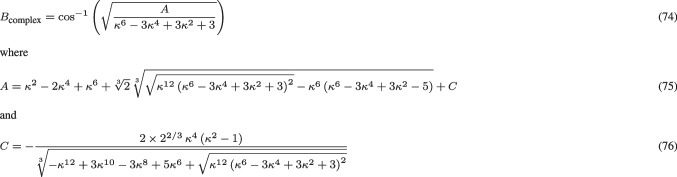
Explicit expression for the orientation selectivity bandwidth *B* for (i) the purely spatial model of complex cells based on affine Gaussian derivatives, as well as for (ii) the velocity-adapted spatio-temporal model of complex cells based on affine Gaussian derivatives

Figure 9 shows the resulting resultants of the orientation selectivity curves obtained in this way, for the first-order models of simple cells, the second-order models of simple cells as well as for the purely spatial model of complex cells and the velocity-adapted models of complex cells.

### The orientation bandwidths *B* of the orientation selectivity curves computed for the affine-Gaussian-derivative-based models of simple and complex cells

To measure the orientation selectivity bandwidth for the first-order simple cells, we solve the equation

81 $$\begin{aligned} \frac{\cos \theta }{\sqrt{\cos ^2 \theta + \kappa ^2 \sin ^2\theta }} = \frac{1}{\sqrt{2}}, \end{aligned}$$

which gives the bandwidth measure

82 $$\begin{aligned} B_{\text{ simple },1} = \cos ^{-1}\left( \frac{\kappa }{\sqrt{\kappa ^2+1}}\right) . \end{aligned}$$

Correspondingly, to determine the orientation selectivity bandwidth for the second-order simple cells, we solve the equation

83 $$\begin{aligned} \frac{\cos ^2 \theta }{\cos ^2 \theta + \kappa ^2 \sin ^2\theta } = \frac{1}{\sqrt{2}}, \end{aligned}$$

which gives the bandwidth measure

84 $$\begin{aligned} B_{\text{ simple },2} = \\ = 2 \tan ^{-1}\left( \sqrt{\frac{-2 \sqrt{2} \, \kappa ^2+2 \sqrt{2} \sqrt{\kappa ^2+\sqrt{2}-1} \, \kappa +\sqrt{2}-2}{\sqrt{2}-2}}\right) . \end{aligned}$$

To determine the corresponding orientation selectivity measure for the purely spatial model of complex cells based on affine Gaussian derivatives, as well as for the velocity-adapted model of complex cells based on affine Gaussian derivatives, we solve the equation

85 $$\begin{aligned} \frac{\left| \cos \theta \right| ^{3/2}}{\left( \cos ^2 \theta + \kappa ^2 \sin ^2\theta \right) ^{3/4}} = \frac{1}{\sqrt{2}}, \end{aligned}$$

which gives the explicit result for the bandwidth measure $B_{\text{ complex }}$ shown in Fig. 10.

**Fig. 11:**
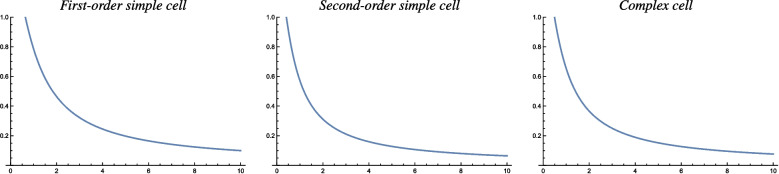
Graphs of the *orientation bandwidth* *B* of the orientation selectivity curves as function of the scale parameter ratio $\kappa$ for the different models of visual receptive fields based on affine Gaussian derivatives. (left) First-order model of simple cell. (middle) Second-order model of complex cell. (right) Purely spatial model of complex cell or velocity-adapted model of complex cell. (Horizontal axes: scale parameter ratio $\kappa$. Vertical axes: orientation bandwidth *B*)

Figure 11 shows the resulting orientation bandwidths of the orientation selectivity curves obtained in this way, for the first-order models of simple cells, the second-order models of simple cells as well as for the purely spatial model of complex cells and the velocity-adapted models of complex cells.

## Perturbation analysis regarding the choices of the angular frequencies and the velocities for the probing sine wave stimuli

In the above theoretical analysis, we have for simplicity of theoretical analysis throughout assumed that the spatial frequency of the probing sine wave has been adapted to the spatial scale parameter of the receptive field. For the velocity-adapted receptive field models, we have additionally assumed that the velocity of the stimulus has been optimally adapted to the velocity parameter of the receptive field.

If one in a biological experiment would, however, not adapt these parameters of the stimuli to their optimal values with regard to each receptive field and each image orientation, one may therefore ask to what extent the above stated theoretical results would be affected by a not optimal match between the parameters of the stimuli to the parameters of the receptive field. To address that question, we will in this section perform different types of perturbation analyses, where the parameters of the stimuli are, thus, not optimally adapted to the parameters of the receptive fields.

### Alternative self-similar selection of the parameters of purely spatial models of simple cells

A first possible generalization to consider of the above mentioned theoretical analysis is by assuming that the angular frequency of the sine wave for probing the receptive field is not necessarily selected from the angular frequency $\hat{\omega }$ for which the receptive field assumes its maximum value, but instead for some scalar constant $\lambda$ times this frequency.

Thus, replacing $\hat{\omega }_{\varphi }$ according to Eq. (33) by

88 $$\begin{aligned} \hat{\omega }_{\varphi } = \frac{\lambda }{\sigma _1 \sqrt{\cos ^2 \theta + \kappa ^2 \sin ^2 \theta }}, \end{aligned}$$

and inserting this value into $A_{\varphi }(\theta , \omega ;\; \sigma _1, \sigma _2)$ according to Eq. (30), and similarly replacing $\hat{\omega }_{\varphi \varphi }$ according to Eq. (38) by

89 $$\begin{aligned} \hat{\omega }_{\varphi \varphi } = \frac{\sqrt{2} \, \lambda }{\sigma _1 \sqrt{\cos ^2 \theta + \kappa ^2 \sin ^2 \theta }}, \end{aligned}$$

and inserting this expression into $A_{\varphi \varphi }(\theta , \omega ;\; \sigma _1, \sigma _2)$ according to Eq. (37), as well as normalizing the resulting orientation selectivity curves to having their peak values equal to 1 for the preferred orientation $\theta = 0$, then gives that the resulting first- and second-order orientation selectivity curves will be independent of the angular frequency scaling factor $\lambda$, and of the forms

90 $$\begin{aligned} {\begin{matrix} A_{\varphi ,\max ,\text{ norm }}(\theta ;\; \kappa ) = \frac{\left| \cos \theta \right| }{\sqrt{\cos ^2 \theta + \kappa ^2 \sin ^2\theta }}, \end{matrix}}\end{aligned}$$

91 $$\begin{aligned} {\begin{matrix} A_{\varphi \varphi ,\max ,\text{ norm }}(\theta ;\; \kappa ) = \frac{\cos ^2 \theta }{\cos ^2 \theta + \kappa ^2 \sin ^2\theta }, \end{matrix}} \end{aligned}$$

that is of similar forms as previously derived in Eqs. (34) and (39). Thus, for this this type of generalization of the probing model, also the compact measures in terms of the resultant *R* according to Eqs. (77) and (78) and the bandwidth *B* according to Eqs. (82) and (84) will be the same.

In this respect, there should be a certain robustness in the shapes of the orientation selectivity curves, as well as concerning the compact resultant and bandwidth measures, with regards to perturbations of the scheme used for quantifying the orientation selectivity properties of the receptive fields.

### Accumulation of orientation selectivity curves without any preferred selection of preferred frequencies for the sine wave probes

A different type of generalization of the probing scheme for the receptive fields would be if we would not perform any adjustment of the angular frequency of the sine wave probe with regard to the frequency selectivity properties of the receptive field for every image orientation of the sine wave probe, but instead keeping the angular frequency the same for all the image orientations.

**Fig. 12:**
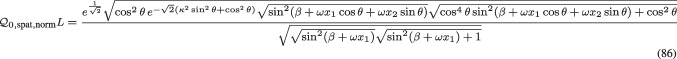
The expression for the oriented spatial quasi-quadrature measure $\mathcal{Q}_{0,\text{ spat },\text{ norm }} L$ in the purely spatial model Eq. (40) of a complex cell, when applied to a sine wave pattern of the form Eq. (27), for $\omega = \root 4 \of {2} \, \lambda /\sigma _1$, *when specifically not adapting the angular frequency of the probing sine wave for each image orientation* $\theta$

For the purely spatial model of receptive fields, then, with $\sigma _2 = \kappa \, \sigma _1$, as well as with $\omega _{\varphi } = \lambda /\sigma _1$ for the first-order simple cell models and with $\omega _{\varphi \varphi } = \sqrt{2} \, \lambda /\sigma _1$ for the second-order simple cell models, as corresponding to the angular frequencies $\omega$ that lead to the maximum response for the preferred direction $\theta = 0$ for the first- and second-order models of simple cells, respectively, according to Eqs. (33) and (38), the amplitude expressions $A_{\varphi }(\theta , \omega ;\; \sigma _1, \sigma _2)$ according to Eq. (30) and $A_{\varphi \varphi }(\theta , \omega ;\; \sigma _1, \sigma _2)$ according to Eq. (37) then assume the forms

92 $$\begin{aligned} {\begin{matrix} A_{\varphi }(\theta \; \kappa , \lambda )&=e^{-\frac{1}{2} (\kappa ^2-1) \, \lambda ^2 \sin ^2 \theta } \, \left| \cos \theta \right| , \end{matrix}}\end{aligned}$$

93 $$\begin{aligned} {\begin{matrix} A_{\varphi \varphi }(\theta ;\; \kappa , \lambda )&= e^{-(\kappa ^2-1) \, \lambda ^2 \sin ^2 \theta } \, \cos ^2\theta . \end{matrix}} \end{aligned}$$

By combining the responses from the first- and the second-order models of simple cells according to Eqs. (29) and (36), respectively, into an idealized model of complex cells according to Eq. (40), for the geometric average $\omega = \sqrt{\omega _{\varphi } \, \omega _{\varphi \varphi }} = \root 4 \of {2} \, \lambda /\sigma _1$ of the preferred angular frequencies for the first- and second-order models of simple cells, respectively, and additionally normalizing the resulting orientation selectivity curve to having its peak value for $\theta = 0$, we obtain an expression for the resulting idealized orientation selectivity curve according to Eq. (86) in Fig. 12.

**Fig. 13:**
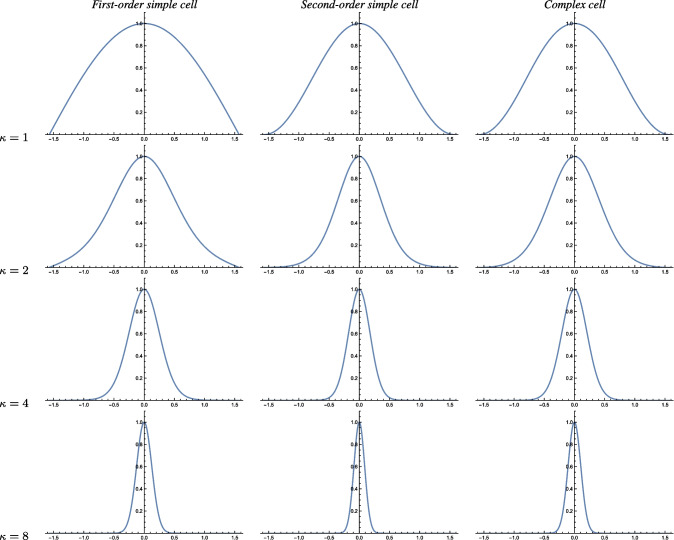
Graphs of the orientation selectivity for *purely spatial models* of (left column) simple cells in terms of first-order directional derivatives of affine Gaussian kernels, (middle column) simple cells in terms of second-order directional derivatives of affine Gaussian kernels and (right column) complex cells in terms of directional quasi-quadrature measures that combine the first- and second-order simple cell responses in a Euclidean way for $C = 1/\sqrt{2}$ and $\lambda = 1$, and shown for different values of the ratio $\kappa$ between the spatial scale parameters in the vertical *vs.* the horizontal directions. In contrast to the previous results in Fig. 3, these curves show the orientation selectivity curves arising *when not adapting the angular frequency of the probing sine wave for each image orientation*. (top row) Results for $\kappa = 1$. (second row) Results for $\kappa = 2$. (third row) Results for $\kappa = 4$. (bottom row) Results for $\kappa = 8$. (Horizontal axes: orientation $\theta \in [-\pi /2, \pi /2]$. Vertical axes: Amplitude of the receptive field response relative to the maximum response obtained for $\theta = 0$)

Figure 13 shows graphs of the idealized models for orientation selectivity curves obtained in this way for the special choice of $\lambda = 1$. As can be seen from the graphs, the qualitative properties of the orientation selectivity curves become more narrow for larger values of the scale parameter ratio $\kappa$. Additionally, larger values of the complementary parameter $\lambda$ will also lead to more narrow orientation selectivity curves, while lower values of the complementary parameter will lead to broader orientation selectivity curves. By varying the parameter $\lambda$ in the model, we can hence predict the effects on the orientation selectivity curve, when using a fixed value of the angular frequency, compared to the situation when the angular frequency of the sine probe would have been adapted to (only) the preferred orientation $\theta = 0$ of the receptive field.

**Fig. 14:**
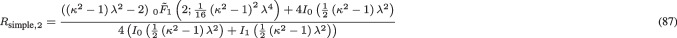
The expression for the resultant of orientation selectivity curve for the second-order idealized model of simple cells, *when specifically not adapting the angular frequency of the probing sine wave for each image orientation* $\theta$. The function $_0\tilde{F}_1(a; z)$ is the hypergeometric function $\operatorname {Hypergeometric0F1Regularized}[a, z]$ in Mathematica, and the function $I_v(z)$ denotes the modified Bessel function of the first kind

**Fig. 15:**
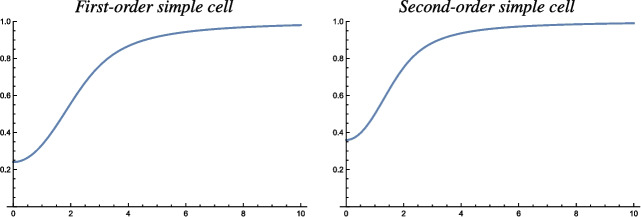
Graphs of the *resultant* *R* of the orientation selectivity curves as function of the scale parameter ratio $\kappa$ for the different models of visual receptive fields based on affine Gaussian derivatives for $\lambda = 1$, *when specifically not adapting the angular frequency of the probing sine wave for each image orientation* $\theta$. (left) First-order model of simple cell. (right) Second-order model of simple cell. (Horizontal axes: scale parameter ratio $\kappa$. Vertical axes: resultant *R*)

The shapes of the resulting idealized orientation selectivity curves do, however, differ somewhat from the idealized orientation selectivity curves in Fig. 3, in that the orientation selectivity curves obtained when not adapting the angular frequency for each image orientation lead to more narrow orientation selectivity properties, compared to the situation when adapting the angular frequency of the sine probe for each angular frequency.

#### Resultant measures for the orientation selectivity curves

By computing the resultant according to Eq. (71) for these orientation selectivity curves, we do furthermore get the resultant for the first-order model of simple cells as

94 $$\begin{aligned} R_{\text{ simple },1} = \frac{2 \, \sqrt{\frac{2}{\pi }} \, e^{-\frac{1}{2} \left( \kappa ^2-1\right) \lambda ^2}}{\sqrt{\kappa ^2-1} \, \lambda \, \operatorname {erf}\left( \frac{\sqrt{\kappa ^2-1} \lambda }{\sqrt{2}}\right) }-\frac{2}{\left( \kappa ^2-1\right) \lambda ^2}+1 \end{aligned}$$

and the resultant for the second-order model of simple cells according to Eq. (87) in Fig. 14.

Figure 15 shows graphs of these curves for the special case of $\lambda = 1$. As can be seen from these graphs, the resulting values of the resultant due to the more narrow orientation selectivity properties resulting from this way of probing the receptive fields confined to larger values of the resultant, compared to the previous results in Fig. 9.

Computing explicit expressions for the resultant of the corresponding idealized model of complex cells is, however, not manageable in closed form. Neither is it manageable to calculate explicit expressions in closed form for the bandwidth descriptors for either the idealized models of simple or complex cells, when using this probing criterion.

### Alternative self-similar selection of the parameters of velocity-adapted spatio-temporal models of simple cells

Concerning corresponding generalizations for a joint spatio-temporal analysis, a first generalization to consider is by assuming that neither the angular frequency $\omega$ of the probing sine wave nor its image velocity *u* are chosen from the angular frequency $\hat{\omega }$ or the image velocity $\hat{u}$, for which the receptive field assumes its maximum value, but instead for some constants $\lambda$ and $\mu$ times these values.

Let us, hence, replace $\hat{\omega }_{\varphi }$ and $\hat{u}_{\varphi }$ according to Eqs. (48) and (49), respectively, by

95 $$\begin{aligned} \hat{\omega }_{\varphi } = \frac{\lambda }{\sigma _1 \sqrt{\cos ^2 \theta + \kappa ^2 \sin ^2 \theta }}, \end{aligned}$$

96 $$\begin{aligned} \hat{u}_{\varphi } = \mu \, v \cos \theta , \end{aligned}$$

and insert these values into $A_{\varphi }(\theta , u, \omega ;\; \sigma _1, \sigma _2, \sigma _t)$ according to Eq. (47), and similarly replace $\hat{\omega }_{\varphi \varphi }$ and $\hat{u}_{\varphi \varphi }$ according to Eqs. (54) and (55), respectively, by

97 $$\begin{aligned} \hat{\omega }_{\varphi \varphi } = \frac{\sqrt{2} \, \lambda }{\sigma _1 \sqrt{\cos ^2 \theta + \kappa ^2 \sin ^2 \theta }}, \end{aligned}$$

98 $$\begin{aligned} \hat{u}_{\varphi \varphi } = \mu \, v \cos \theta . \end{aligned}$$

and insert these expressions into $A_{\varphi \varphi }(\theta , u, \omega ;\; \sigma _1, \sigma _2, \sigma _t)$ according to Eq. (53), which then, after normalizing the resulting orientation selectivity curves to having their peak values equal to 1, leads to

99 $$\begin{aligned} A_{\varphi ,\max ,\text{ norm }}(\theta ;\; \sigma _1, \sigma _t, \kappa , \mu ) = \\ = \frac{\left| \cos \theta \right| \, \sqrt{(\mu -1)^2 \, \sigma _t^2 \, v^2+\sigma _1^2}}{\sqrt{\kappa ^2 \, \sigma _1^2 \sin ^2\theta +\cos ^2\theta \left( (\mu -1)^2 \, \sigma _t^2 \, v^2+\sigma _1^2\right) }}, \end{aligned}$$

100 $$\begin{aligned} A_{\varphi \varphi ,\max ,\text{ norm }}(\theta ;\; \sigma _1, \sigma _t, \kappa , \mu ) = \\ = \frac{\cos ^2\theta \left( (\mu -1)^2 \, \sigma _t^2 \, v^2+\sigma _1^2\right) }{\kappa ^2 \, \sigma _1^2 \sin ^2\theta +\cos ^2(\theta ) \left( (\mu -1)^2 \, \sigma _t^2 \, v^2+\sigma _1^2\right) }. \end{aligned}$$

Notably, these orientation selectivity curves are, although independent of the frequency scaling factor $\lambda$, for general values of the velocity scaling factor $\mu$, in addition to a dependency on the scale parameter ratio $\kappa$, also strongly dependent on both the spatial and temporal scale parameters $\sigma _1$ and $\sigma _t$ as well as the velocity parameter *v* in the receptive field model.

If we restrict the velocity scaling factor $\mu$ to $\mu = 1$, however, then these orientation selectivity curves reduce to the forms previously derived when also selecting the angular frequency of the sine wave probe to the value $\hat{\omega }$, that leads to the strongest response for the receptive field, of the forms

103 $$\begin{aligned} {\begin{matrix} A_{\varphi ,\max ,\text{ norm }}(\theta ;\; \kappa )&= \frac{\left| \cos \theta \right| }{\sqrt{\cos ^2\theta + \kappa ^2 \sin ^2\theta }}, \end{matrix}}\end{aligned}$$

104 $$\begin{aligned} {\begin{matrix} A_{\varphi \varphi ,\max ,\text{ norm }}(\theta ;\; \kappa )&= \frac{\cos ^2\theta }{\cos ^2\theta + \kappa ^2 \sin ^2\theta }. \end{matrix}} \end{aligned}$$

In this way, for the special case of choosing the velocity scaling factor as $\mu = 1$, this spatio-temporal analysis generalizes the results of the purely spatial analysis in Sect. 7.1.

For values of the velocity scaling factor $\mu \ne 1$, the previous role of the square of the scale parameter ratio $\kappa ^2$ in the idealized shape of the orientation selectivity curve is, however, now replaced by the following parameter

105 $$\begin{aligned} \tilde{\kappa }^2 = \frac{\kappa ^2 \, \sigma _1^2}{(\mu -1)^2 \, \sigma _t^2 \, v^2+\sigma _1^2} = \frac{\kappa ^2}{1 + (\mu -1)^2 \, \left( \frac{\sigma _t \, v}{\sigma _1}\right) ^2}, \end{aligned}$$

where the dimensionless ratio

106 $$\begin{aligned} \tilde{v} = \frac{\sigma _t}{\sigma _1} \, v \end{aligned}$$

is a scale-normalized velocity parameter of the receptive field, such that the shapes of the orientation selectivity curves in the spatio-temporal case then assume the forms

107 $$\begin{aligned} {\begin{matrix} A_{\varphi ,\max ,\text{ norm }}(\theta ;\; \tilde{\kappa })&= \frac{\left| \cos \theta \right| }{\sqrt{\cos ^2\theta + \tilde{\kappa } ^2 \sin ^2\theta }}, \end{matrix}}\end{aligned}$$

108 $$\begin{aligned} {\begin{matrix} A_{\varphi \varphi ,\max ,\text{ norm }}(\theta ;\; \tilde{\kappa })&= \frac{\cos ^2\theta }{\cos ^2\theta + \tilde{\kappa } ^2 \sin ^2\theta }. \end{matrix}} \end{aligned}$$

Thus, for non-matching values of the parameters of the sine wave probe to the optimal values with regard to any specific receptive field, the shapes of the orientation selectivity curves will be affected by the degree of such a mismatch between the parameters of the probing sine wave in relation to the parameters of the receptive field.

### Accumulation of orientation selectivity curves without any preferred selection of preferred frequencies or velocities for the sine wave probes

Alternatively, we can also consider a situation, when neither the angular frequency or the image velocity of the sine wave probe is adapted to the properties of the receptive field.

**Fig. 16:**
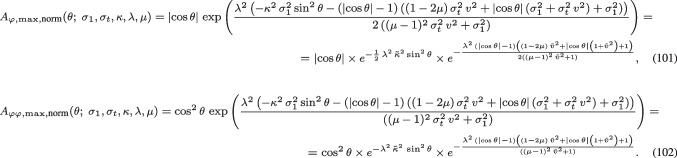
The expressions for the orientation selectivity curve for the first- and second-order velocity-adapted spatio-temporal models of simple cells, *when specifically not adapting the angular frequency of the probing sine wave for each image orientation* $\theta$

Let us therefore introduce an angular frequency scaling parameter $\lambda$ as well as a velocity scaling parameter $\mu$ normalized such that the special values $\lambda = 1$ and $\mu = 1$ would correspond to the same experimental situation as if the angular frequency $\omega$ as well as the image velocity *u* would be adapted to correspond to the maximum possible value of the receptive field response for the preferred orientation $\theta = 0$.

Then, for the first-order model of the receptive field, we parameterize the angular frequency and the image velocity according to Eqs. (48) and (49)

109 $$\begin{aligned} {\begin{matrix} \omega _{\varphi } = \frac{\lambda }{\sqrt{(\mu -1)^2 \, \sigma _t^2 \, v^2+\sigma _1^2}}, \end{matrix}}\end{aligned}$$

110 $$\begin{aligned} {\begin{matrix} u_{\varphi } = \mu \, v, \end{matrix}} \end{aligned}$$

while we for the second-order model of the receptive field we parameterize the angular frequency and the image velocity according to Eqs. (54) and (55)

111 $$\begin{aligned} {\begin{matrix} \omega _{\varphi } = \frac{\sqrt{2} \, \lambda }{\sqrt{(\mu -1)^2 \, \sigma _t^2 \, v^2+\sigma _1^2}}, \end{matrix}}\end{aligned}$$

112 $$\begin{aligned} {\begin{matrix} u_{\varphi } = \mu \, v, \end{matrix}} \end{aligned}$$

which, after insertion into $A_{\varphi }(\theta , u, \omega ;\; \sigma _1, \sigma _2, \sigma _t)$ according to Eq. (47) and $A_{\varphi \varphi }(\theta , u, \omega ;\; \sigma _1, \sigma _2, \sigma _t)$ according to Eq. (53), leads to the expressions for the orientation selectivity curves for the first- and second-order receptive fields shown in Eqs. (101) and (102) in Fig. 16, if we also normalize these curves to having their peak values equal to 1.

From a more detailed examination of this expression, when reformulated in terms of the transformed scale parameter ratio $\tilde{\kappa }$ according to Eq. (105) and the dimensionless scale-normalized velocity parameter $\tilde{v}$ according to Eq. (106), we can note that the resulting orientation selectivity curves constitue products of (i) a cosine function raised to the order of differentiation, (ii) a negative exponential in terms of the transformed scale parameter ratio $\tilde{\kappa }$ and (iii) a negative exponential in terms of the dimensionless scale-normalized velocity parameter $\tilde{v}$, where the complementary frequency scaling factor $\lambda$ and the complementary velocity scaling factor $\mu$ are also dimensionless.

For this spatio-temporal analysis, the shape of the orientation selectivity curves will therefore be strongly dependent on the relationships between the parameters of the probing sine wave and the parameters of the receptive field. From such a perspective, a conceptual advantage of the probing method used in the previous theoretical analysis in Sect. 4 is therefore that, by adapting the parameters of the sine wave probe to the their optimal values for each probing situation, we can derive characteristic properties of the orientation selectivity properties of the receptive field, that constitute fully characteristic properties of that receptive field, and not depending on any otherwise *a priori* unknown mismatch between the parameters of the sine wave probe in relation to the parameters of the receptive field.

## Summary and discussion

We have presented an in-depth theoretical analysis of the orientation selectivity properties for the spatial and spatio-temporal receptive fields according to the generalized Gaussian derivative model for visual receptive fields, summarized in Sects. 3.1 and 3.3. This model for visual receptive fields has been previously derived in an axiomatic manner, from symmetry properties of the environment, in combination with requirements of internal consistency between image representations over multiple spatial and temporal scales. This model has notably also been demonstrated to well capture the properties of biological simple cells in the primary visual cortex. Building upon that theory for linear receptive fields, we have also analysed the orientation selectivity for (some of them new) non-linear models of complex cells, summarized in Sects. 3.2 and 3.4, based on energy models that combine the output from such models for simple cells for different orders of spatial differentiation.

Specifically, we have in Sect. 4, with the details concerning the analysis of the space-time separable spatio-temporal models in Appendix A.1, analyzed how the orientation selectivity depends on a scale parameter ratio $\kappa$, between the scale parameters in the image orientations perpendicular to *vs.* parallel with the preferred orientation of the receptive fields. Explicit expressions for the resulting orientation selectivity curves have been derived, based on closed form theoretical analysis, and it has been shown that, for all these models of visual receptive fields, the degree of orientation selectivity becomes more narrow with increasing values of the scale parameter ratio $\kappa$. Additionally, we have in Sect. 6 derived closed-form expressions for the resultant and the bandwidth of the orientation selectivity curves, which can be used for interpreting previous compact characterizations of neurophysiological measurements of orientation selectivity to parameters in the idealized models for visual receptive fields.

To compare the theoretical predictions obtained based on the affine Gaussian derivative model for visual receptive fields, to what would be obtained from basing the theoretical analysis on other types of receptive field models, we have furthermore in Sect. 5 presented a detailed orientation selectivity analysis for an an affine Gabor model of visual receptive fields. The results obtained from the affine Gabor model are consistent with the results obtained from the affine Gaussian derivative model, in the sense that the orientation selectivity becomes more narrow, as we widen the receptive fields in the direction perpendicular to preferred orientation of the receptive fields.

The results from the affine Gabor model do, on the other hand, also differ from the results obtained from the affine Gaussian derivative model, in the respect that the parameter space of the affine Gabor model comprises one more degree of freedom, compared to the parameter space of the affine Gaussian derivative model. Specifically, a variability along that additional degree of freedom does also strongly affect the orientation selectivity of the receptive fields according to the affine Gabor model. The connections between the degree of orientation selectivity and the elongation of the receptive fields obtained from the affine Gaussian derivative model are in this respect more specific than for the affine Gabor model. This points to a both a qualitative similarity and a qualitative difference between the affine Gaussian derivative model and the affine Gabor model.

We have also in Appendix A.2 shown that a generalized version of the affine Gabor model also supports affine covariance, as the affine Gaussian derivative model does.

Concerning extensions of the approach, we have in the present treatment regarding receptive fields according to the generalized Gaussian derivative model for visual receptive fields, limited ourselves to receptive fields corresponding to only first- and second-order derivatives over the spatial domain. Modelling results by Young (1987) have, however, demonstrated that receptive fields up to fourth order of spatial differentiation may be present in the primary visual cortex. It is straightforward to extend our analysis to third- and fourth-order directional derivatives, which would then give other closed-form expressions for the orientation selectivity of the receptive fields (and more narrow than the orientation selectivity for first- and second-order derivatives). Models of complex cells involving third- and fourth-order spatial derivatives could also be formulated and be theoretically analyzed, although experimental support for such extended models of complex cells may currently not be available.

A more conceptual way of extending the modelling work would also be to incorporate a complementary spatial smoothing step in model of complex cells, as used in (Lindeberg 2020 Section 5). In the models for complex cells used in the theoretical analysis in this paper, the responses of first- and second-order spatial derivative based receptive fields have been throughout combined in a pointwise manner, over both space and time. A more general approach would, however, be to perform weighted integration of such pointwise contributions over a wider support region over space and time, with the size over the spatial image domain and the duration over the temporal domain proportional to the local spatial and temporal scales at which the spatial and temporal derivatives are computed. In the present treatment, we have, however, not extended the complex cell models in that way, although it could be well motivated, mainly to simplify the treatment, and to limit the complexity of the theoretical analysis. If the orientation selectivity curves obtained from theoretical analysis are to be fitted to data from actual neurophysiological measurements, it does, however, seem advisable to complement the models for complex cells by explicit spatial integration, as done in Equation (46) in Lindeberg (2020).

Concerning the spatio-temporal models of the receptive fields, we have also limited ourselves to performing a non-causal temporal analysis, where the temporal smoothing kernels are 1-D Gaussian kernels. To perform a corresponding time-causal temporal analysis, based on using the time-causal limit kernel Eq. (15) for temporal smoothing, one can perform a Fourier analysis to determine how the probing sine wave will be affected, using the closed-form expression for the Fourier transform of the time-causal limit kernel, in a similar way as in the temporal scale selection analysis in (Lindeberg 2017 Section 5.2).

Concerning the way that the orientation selectivity curves are defined, we have in the above theoretical analysis for the affine Gaussian derivative model throughout^8^ in Sect. 4 assumed that the wavelength $\hat{\omega }$ (and for the spatio-temporal analysis also the image velocity $\hat{u}$) of the probing sine wave is optimized for each image orientation separately. Another possibility is to instead assume that the wavelength is only optimized for the preferred orientation of the receptive field only, and then held constant for all the other inclination angles $\theta$. Changing the probing method in such a way would then change the shapes of the orientation selectivity curves, but could be easily performed, based on the principles for theoretical analysis outlined in the above treatment.

In Sect. 7 we have specifically presented a complementary theoretical analysis of such alternative probing methods, for both the purely spatial models and the velocity-adapted spatio-temporal models of the receptive fields, and demonstrated how such modelling may lead to more narrow orientation selectivity curves, compared to the mainly studied probing method, where the angular frequency of the sine wave probe is adapted to each orientation of the sine wave stimuli.

One could possibly also consider extending the analysis to different values of the scale normalization powers $\gamma$ and $\varGamma$, than using the maximally scale invariant choices $\gamma = 1$ and $\varGamma = 0$ used for simplicity in this treatment. Then, however, some type of post-normalization may, however, also be necessary, to make it possible to appropriately compare receptive field responses between multiple spatial and temporal scales, which otherwise are perfectly comparable when using $\gamma = 1$ and $\varGamma = 0$.

The complementary analysis in Sects. 7.1 and 7.3, where the frequency of the probing sine wave is not selected from the maximum over the angular frequency, but instead from a constant $\lambda$ times that angular frequency value, demonstrates that in an experimental situation where the angular frequency is adapted to each image orientation, the resulting normalized orientation selectivity curves are invariant under variations of the parameter $\lambda$. In the case when the angular frequency of the sine wave probe is not adapted to each stimulus orientation, as studied in Sects. 7.2 and 7.4, the introduction of a corresponding parameter $\lambda$ does, however, strongly influence the shapes of the orientation selectivity curves, and then also in close interaction with the scale parameter ratio $\kappa$. Additionally, regarding the complementary analysis of velocity-adapted spatio-temporal receptive fields in Sects. 7.3 and 7.4, a mismatch between the velocity of the probing sine wave in relation to the preferred velocity of the receptive field may also strongly affect the shapes of the orientation selectivity curves.

A take-home message from this complementary analysis is that the shapes of the orientation selectivity curves may, in these respects, be strongly dependent on the parameters of the sine wave stimuli, that are used for probing the orientation selectivity properties of the receptive fields, unless adapting the parameters of the sine wave probe to maximize the response properties of the receptive field.

The proposed probing method in Sect. 4, where the angular frequency of the sine wave probe is for each image orientation determined from the angular frequency that maximizes the response over the angular frequencies, and for velocity-adapted spatio-temporal receptives the image velocity of the stimulus probe is also determined from the image velocity that maximizes the response over the image velocities, is in this respect very special, in the sense that the resulting orientation selectivity do not risk being hampered by an otherwise possible mismatch between the parameters of the stimulus probe in relation to the inherent parameters of the receptive field.

Concerning possible limitations of the approach, it should be emphasized that the models for visual receptive fields in the generalized Gaussian derivative model are highly idealized. They have been derived from mathematical analysis based on idealized theoretical assumptions, regarding symmetry properties of the environment, while they have not been quantitatively adjusted to the receptive field shapes of actual biological neurons. Hence, the results from the presented theoretical analysis should be interpreted as such, as the results of a maximally idealized model, and not with specific aims of providing a numerically accurate representation of actual biological neurons. The receptive field models also constitute pure feed-forward models with no feed-back mechanisms, which are otherwise known to be important in biological vision.

**Fig. 17:**
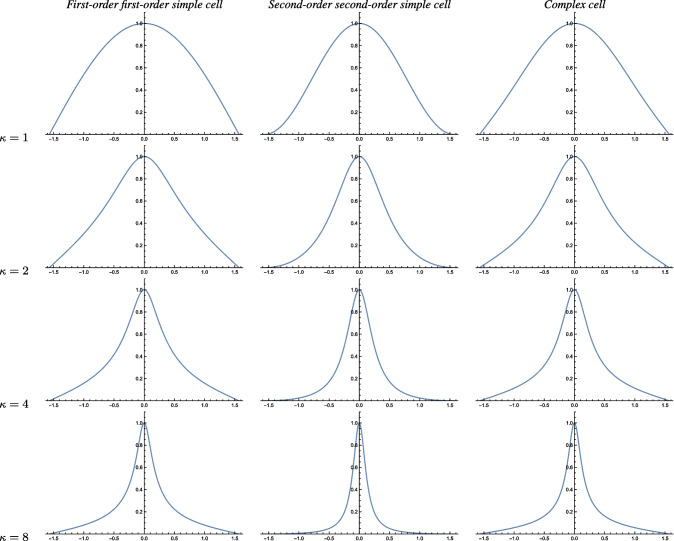
Graphs of the orientation selectivity for *space-time separable spatio-temporal models* of (left column) simple cells in terms of first-order directional derivatives of affine Gaussian kernels combined with first-order temporal derivatives of temporal Gaussian kernels, (middle column) simple cells in terms of second-order directional derivatives of affine Gaussian kernels combined with second-order temporal derivatives of temporal Gaussian kernels and (right column) complex cells in terms of directional quasi-quadrature measures that combine the first- and second-order simple cell responses in a Euclidean way for $C_{\varphi } = 1/\sqrt{2}$, and $C_t = 1/\sqrt{2}$ shown for different values of the ratio $\kappa$ between the spatial scale parameters in the vertical *vs.* the horizontal directions. Observe how the degree of orientation selectivity varies strongly depending on the eccentricity $\epsilon = 1/\kappa$ of the receptive fields. (top row) Results for $\kappa = 1$. (second row) Results for $\kappa = 2$. (third row) Results for $\kappa = 4$. (bottom row) Results for $\kappa = 8$. (Horizontal axes: orientation $\theta \in [-\pi /2, \pi /2]$. Vertical axes: Amplitude of the receptive field response relative to the maximum response obtained for $\theta = 0$)

In view of such a background, it is, however, highly interesting to see how well the derived orientation selectivity curves in Figs. 3, 5 and 17 reflect the qualitative shapes of the biologically established orientation selectivity curves recorded in the primary visual cortex by Nauhaus et al. (2008), as reflected in the qualitative comparison to those results in our companion paper (Lindeberg, 2024a).

Additionally, it is interesting to note that corresponding closed-form calculations of the resultant, for the orientation selectivity curves for the idealized models of simple cells based on affine Gaussian derivatives, lead to predictions that are qualitatively very similar to the experimentally obtained histograms of the resultant for the orientation selectivity curves, accumulated for biological simple cells by Goris et al. (2015), if extended to models of simple cells in terms of affine Gaussian derivatives up to order 4, see Lindeberg (2024a) for further details.

It is also known that the distinction between simple and complex cells may not be as distinct as proposed in the initial work by Hubel and Wiesel, where there could instead be a scale of gradual transitions between simple and complex cells, see Alonso and Martinez (1998); Mechler and Ringach (2002); Li et al. (2015) for treatments with different views on this topic. Considering that the forms of the derived orientation selectivity are often similar for our models of simple and complex cells (as summarized in Table 1), one may speculate that the presented results could be relevant with respect to such a wider contextual background.

It may also be worth stating that we do not in any way exclude the possibility of deriving corresponding prediction results for other possible models of receptive fields, or for models of neural mechanisms that lead to the formation of visual receptive fields. A motivation, for in this paper restricting the analysis in this paper to the idealized receptive fields according to the generalized Gaussian derivative model and to the idealized receptive fields according to the Gabor model, is that these models may be regarded the most commonly used and well-established idealized functional models for linear receptive fields in the primary visual cortex. These receptive field models do specifically reasonably well reproduce the qualitative shape of many receptive fields in the primary visual cortex, as have been established by neurophysiological measurements. The formulation of possible Gabor models for spatio-temporal receptive fields does, however, appear as a more open topic. Therefore, we have here restricted the analysis of the affine Gabor model to purely spatial receptive fields.

Considering also that the relationship between the orientation selectivity and the degree of elongation of the receptive fields is more direct for the affine-Gaussian-derivative-based model than for the affine Gabor model, an overall conclusion from this work is therefore that the affine-Gaussian-derivative-based model for visual receptive fields is conceptually much easier to analyze with regard to orientation selectivity properties than the affine Gabor model.

Based on the theoretical analysis presented in this paper, we propose that very interesting work could be done to match the output from measurements of the orientation selectivity properties of biological receptive fields to theoretical models of their functional properties.

## Supplementary Information

Below is the link to the electronic supplementary material.

Supplementary file 1 (zip 821 KB)

## Data Availability

No datasets were generated or analysed during the current study.

## A Appendix

### A.1 Analysis for space-time separable models of spatio-temporal receptive fields

To investigate the directional selectivity of our spatio-temporal models for simple and complex cells, we will analyze their response properties to a moving sine wave of the form

113 $$\begin{aligned} f(x_1, x_2, t) = \\ = \sin \left( \omega \cos (\theta ) \, (x_1 - u_1 t) + \omega \sin (\theta ) \, (x_2 - u_2 t) + \beta \right) , \end{aligned}$$

where we choose the velocity vector $(u_1, u_2)^T$ parallel to the inclination angle $\theta$ of the grating according to $(u_1, u_2)^T = (u \cos \theta , u \sin \theta )^T$, which, in turn, implies the form

114 $$\begin{aligned} f(x_1, x_2, t) = \sin \left( \omega \cos (\theta ) \, x_1 + \omega \sin (\theta ) \, x_2 - u \, t + \beta \right) . \end{aligned}$$

Let us initially perform such an analysis for space-time separable models of simple and complex cells, in which the velocity vector *v* in the spatio-temporal receptive field models is set to zero.

For simplicity, we initially perform the theoretical analysis for non-causal spatio-temporal receptive field models, where the temporal components are given as scale-normalized temporal derivatives of 1-D temporal Gaussian kernels.

In the following, we will name our models of spatio-temporal receptive fields according to the orders of differentiation with respect to space and time.

#### A.1.1 First-order first-order simple cell

Consider a space-time separable receptive field corresponding to a *first-order* scale-normalized Gaussian derivative with scale parameter $\sigma _1$ in the horizontal $x_1$-direction, a zero-order Gaussian kernel with scale parameter $\sigma _2$ in the vertical $x_2$-direction, and a *first-order* scale-normalized Gaussian derivative with scale parameter $\sigma _t$ in the temporal direction, corresponding to $\varphi = 0$, $v = 0$, $\varSigma _0= \operatorname {diag}(\sigma _1^2, \sigma _2^2)$, $m = 1$ and $n = 1$ in Eq. (13):

115 $$\begin{aligned} {\begin{matrix}&T_{0,t,\text{ norm }}(x_1, x_2, t;\; \sigma _1, \sigma _2, \sigma _t) = \end{matrix}}\nonumber \\ {\begin{matrix}&= \frac{\sigma _1 \sigma _t}{(2 \pi )^{3/2} \, \sigma _1 \sigma _2 \sigma _t} \, \partial _{x_1} \partial _{t} \left( e^{-x_1^2/2\sigma _1^2 - x_2^2/2 \sigma _2^2 - t^2/2\sigma _t^2} \right) \end{matrix}}\nonumber \\ {\begin{matrix}&= \frac{x_1 t}{(2 \pi )^{3/2} \, \sigma _1^2 \sigma _2 \sigma _t^2} \, e^{-x_1^2/2\sigma _1^2 - x_2^2/2 \sigma _2^2 - t^2/2\sigma _t^2}. \end{matrix}} \end{aligned}$$

The corresponding receptive field response is then, after solving the convolution integral in Mathematica,

116 $$\begin{aligned} {\begin{matrix} L_{0,t,\text{ norm }}(x_1, x_2, t;\; \sigma _1, \sigma _2, \sigma _t) = \end{matrix}}\nonumber \\ {\begin{matrix}&= \int _{\xi _1 = -\infty }^{\infty } \int _{\xi _2 = -\infty }^{\infty } \int _{\zeta = -\infty }^{\infty } T_{0,t,\text{ norm }}(\xi _1, \xi _2, \zeta ;\; \sigma _1, \sigma _2, \sigma _t) \end{matrix}}\nonumber \\ {\begin{matrix}&{\hspace{85.0pt}} \times f(x_1 - \xi _1, x_2 - \xi _2, t - \zeta ) \, d \xi _1 \xi _2 d\zeta \end{matrix}}\nonumber \\ {\begin{matrix}&= \omega ^2 \, \sigma _1 \, \sigma _t \, u \cos (\theta ) \, e^{-\frac{1}{2} \omega ^2 (\sigma _1^2 \cos ^2 \theta + \sigma _2^2 \sin ^2 \theta + \sigma _t^2 u^2)} \end{matrix}}\nonumber \\ {\begin{matrix}&{\hspace{15.0pt}} \times \sin \left( \omega \cos (\theta ) \, x_1 + \omega \sin (\theta ) \, x_2 - u \, t + \beta \right) , \end{matrix}} \end{aligned}$$

*i.e.*, a sine wave with amplitude

117 $$\begin{aligned} A_{\varphi ,t}(\theta , u, \omega ;\; \sigma _1, \sigma _2, \sigma _t) = \\ = \omega ^2 \, \sigma _1 \, \sigma _t \, u \left| \cos \theta \right| \, e^{-\frac{1}{2} \omega ^2 (\sigma _1^2 \cos ^2 \theta + \sigma _2^2 \sin ^2 \theta + \sigma _t^2 u^2)}. \end{aligned}$$

This expression first increases and then decreases with respect to both the angular frequency $\omega$ and the velocity *u* of the sine wave. Selecting the values of $\hat{\omega }$ and $\hat{u}$ at which this expression assumes its maximum over $\omega$ and *u*

118 $$\begin{aligned} \hat{\omega }_{\varphi ,t} = \frac{1}{\sigma _1 \sqrt{\cos ^2 \theta + \kappa ^2 \sin ^2 \theta }}, \end{aligned}$$

119 $$\begin{aligned} \hat{u}_{\varphi ,t} = \frac{\sigma _1}{\sigma _t} \sqrt{\cos ^2 \theta + \kappa ^2 \sin ^2 \theta }, \end{aligned}$$

and again reparameterizing the other spatial scale parameter $\sigma _2$ as $\sigma _2 = \kappa \, \sigma _1$, gives that the maximum amplitude measure over spatial and temporal scales is

120 $$\begin{aligned} A_{\varphi ,t,\max }(\theta ;\; \kappa ) = \frac{\left| \cos \theta \right| }{e \sqrt{\cos ^2 \theta + \kappa ^2 \sin ^2\theta }}. \end{aligned}$$

Note that again this directional selectivity measure is independent of the spatial scale parameter $\sigma _1$ as well as independent of the temporal scale parameter $\sigma _t$, because of the scale-invariant property of scale-normalized derivatives for scale normalization power $\gamma = 1$.

The left column in Fig. 17 shows the result of plotting the measure $A_{\varphi ,t,\max }(\theta ;\; \kappa )$ of the orientation selectivity as function of the inclination angle $\theta$ for a few values of the scale parameter ratio $\kappa$, with the values rescaled such that the peak value for each graph is equal to 1. As we can see from the graphs, as for the previous purely spatial model of the receptive fields, the degree of orientation selectivity increases strongly with the value of $\kappa$.

#### A.1.2 First-order second-order simple cell

Consider a space-time separable receptive field corresponding to a *first-order* scale-normalized Gaussian derivative with scale parameter $\sigma _1$ in the horizontal $x_1$-direction, a zero-order Gaussian kernel with scale parameter $\sigma _2$ in the vertical $x_2$-direction, and a *second-order* scale-normalized Gaussian derivative with scale parameter $\sigma _t$ in the temporal direction, corresponding to $\varphi = 0$, $v = 0$, $\varSigma _0 = \operatorname {diag}(\sigma _1^2, \sigma _2^2)$, $m = 1$ and $n = 2$ in Eq. (13):

121 $$\begin{aligned} {\begin{matrix}&T_{0,tt,\text{ norm }}(x_1, x_2, t;\; \sigma _1, \sigma _2, \sigma _t) = \end{matrix}}\nonumber \\ {\begin{matrix}&= \frac{\sigma _1 \sigma _t^2}{(2 \pi )^{3/2} \, \sigma _1 \sigma _2 \sigma _t} \, \partial _{x_1} \partial _{tt} \left( e^{-x_1^2/2\sigma _1^2 - x_2^2/2 \sigma _2^2 - t^2/2\sigma _t^2} \right) \end{matrix}}\nonumber \\ {\begin{matrix}&= - \frac{x_1 (t^2 - \sigma _t^2)}{(2 \pi )^{3/2} \, \sigma _1^2 \sigma _2 \sigma _t^3} \, e^{-x_1^2/2\sigma _1^2 - x_2^2/2 \sigma _2^2 - t^2/2\sigma _t^2}. \end{matrix}} \end{aligned}$$

The corresponding receptive field response is then, after solving the convolution integral in Mathematica,

122 $$\begin{aligned} {\begin{matrix} L_{0,tt,\text{ norm }}(x_1, x_2, t;\; \sigma _1, \sigma _2, \sigma _t) = \end{matrix}}\nonumber \\ {\begin{matrix}&= \int _{\xi _1 = -\infty }^{\infty } \int _{\xi _2 = -\infty }^{\infty } \int _{\zeta = -\infty }^{\infty } T_{0,tt,\text{ norm }}(\xi _1, \xi _2, \zeta ;\; \sigma _1, \sigma _2, \sigma _t) \end{matrix}}\nonumber \\ {\begin{matrix}&{\hspace{85.0pt}} \times f(x_1 - \xi _1, x_2 - \xi _2, t - \zeta ) \, d \xi _1 \xi _2 d\zeta \end{matrix}}\nonumber \\ {\begin{matrix}&= - \omega ^3 \, \sigma _1 \, \sigma _t^2 \, u^2 \cos (\theta ) \, e^{-\frac{1}{2} \omega ^2 (\sigma _1^2 \cos ^2 \theta + \sigma _2^2 \sin ^2 \theta + \sigma _t^2 u^2)} \end{matrix}}\nonumber \\ {\begin{matrix}&{\hspace{15.0pt}} \times \cos \left( \omega \cos (\theta ) \, x_1 + \omega \sin (\theta ) \, x_2 - u \, t + \beta \right) , \end{matrix}} \end{aligned}$$

*i.e.*, a cosine wave with amplitude

123 $$\begin{aligned} A_{\varphi ,tt}(\theta , u, \omega ;\; \sigma _1, \sigma _2, \sigma _t) = \\ = \omega ^3 \, \sigma _1 \, \sigma _t^2 \, u^2 \left| \cos \theta \right| \, e^{-\frac{1}{2} \omega ^2 (\sigma _1^2 \cos ^2 \theta + \sigma _2^2 \sin ^2 \theta + \sigma _t^2 u^2)}. \end{aligned}$$

This entity assumes its maximum over the angular frequency $\omega$ and the image velocity *u* at

124 $$\begin{aligned} \hat{\omega }_{\varphi ,tt} = \frac{1}{\sigma _1 \sqrt{\cos ^2 \theta + \kappa ^2 \sin ^2 \theta }}, \end{aligned}$$

125 $$\begin{aligned} \hat{u}_{\varphi ,tt} = \frac{\sqrt{2} \sigma _1}{\sigma _t} \sqrt{\cos ^2 \theta + \kappa ^2 \sin ^2 \theta }, \end{aligned}$$

and again reparameterizing the other spatial scale parameter $\sigma _2$ as $\sigma _2 = \kappa \, \sigma _1$, gives that the maximum amplitude measure over spatial and temporal scales is

126 $$\begin{aligned} A_{\varphi ,tt,\max }(\theta ;\; \kappa ) = \frac{2 \left| \cos \theta \right| }{e^{3/2} \sqrt{\cos ^2 \theta + \kappa ^2 \sin ^2\theta }}, \end{aligned}$$

*i.e.*, of a similar form as the previous measure $A_{\varphi ,tt,\max }(\theta ;\;$ $\kappa )$, while being multiplied by another constant.

#### A.1.3 Second-order first-order simple cell

Consider a space-time separable receptive field corresponding to a *second-order* scale-normalized Gaussian derivative with scale parameter $\sigma _1$ in the horizontal $x_1$-direction, a zero-order Gaussian kernel with scale parameter $\sigma _2$ in the vertical $x_2$-direction, and a *first-order* scale-normalized Gaussian derivative with scale parameter $\sigma _t$ in the temporal direction, corresponding to $\varphi = 0$, $v = 0$, $\varSigma _0 = \operatorname {diag}(\sigma _1^2, \sigma _2^2)$, $m = 2$ and $n = 1$ in Eq. (13):

127 $$\begin{aligned} {\begin{matrix}&T_{00,t,\text{ norm }}(x_1, x_2, t;\; \sigma _1, \sigma _2, \sigma _t) = \end{matrix}}\nonumber \\ {\begin{matrix}&= \frac{\sigma _1^2 \sigma _t}{(2 \pi )^{3/2} \, \sigma _1 \sigma _2 \sigma _t} \, \partial _{x_1 x_1} \partial _{t} \left( e^{-x_1^2/2\sigma _1^2 - x_2^2/2 \sigma _2^2 - t^2/2\sigma _t^2} \right) \end{matrix}}\nonumber \\ {\begin{matrix}&= - \frac{(x_1^2 - \sigma _1^2) t}{(2 \pi )^{3/2} \, \sigma _1^3 \sigma _2 \sigma _t^2} \, e^{-x_1^2/2\sigma _1^2 - x_2^2/2 \sigma _2^2 - t^2/2\sigma _t^2}. \end{matrix}} \end{aligned}$$

The corresponding receptive field response is then, after solving the convolution integral in Mathematica,

128 $$\begin{aligned} {\begin{matrix} L_{00,t,\text{ norm }}(x_1, x_2, t;\; \sigma _1, \sigma _2, \sigma _t) = \end{matrix}}\nonumber \\ {\begin{matrix}&= \int _{\xi _1 = -\infty }^{\infty } \int _{\xi _2 = -\infty }^{\infty } \int _{\zeta = -\infty }^{\infty } T_{00,t,\text{ norm }}(\xi _1, \xi _2, \zeta ;\; \sigma _1, \sigma _2, \sigma _t) \end{matrix}}\nonumber \\ {\begin{matrix}&{\hspace{85.0pt}} \times f(x_1 - \xi _1, x_2 - \xi _2, t - \zeta ) \, d \xi _1 \xi _2 d\zeta \end{matrix}}\nonumber \\ {\begin{matrix}&= -\omega ^3 \, \sigma _1^2 \, \sigma _t \, u \cos ^2 (\theta ) \, e^{-\frac{1}{2} \omega ^2 (\sigma _1^2 \cos ^2 \theta + \sigma _2^2 \sin ^2 \theta + \sigma _t^2 u^2)} \end{matrix}}\nonumber \\ {\begin{matrix}&{\hspace{15.0pt}} \times \cos \left( \omega \cos (\theta ) \, x_1 + \omega \sin (\theta ) \, x_2 - u \, t + \beta \right) , \end{matrix}} \end{aligned}$$

*i.e.*, a cosine wave with amplitude

129 $$\begin{aligned} A_{\varphi \varphi ,t}(\theta , u, \omega ;\; \sigma _1, \sigma _2, \sigma _t) = \\ = \omega ^3 \, \sigma _1^2 \, \sigma _t \, u \cos ^2 \theta \, e^{-\frac{1}{2} \omega ^2 (\sigma _1^2 \cos ^2 \theta + \sigma _2^2 \sin ^2 \theta + \sigma _t^2 u^2)}. \end{aligned}$$

This entity assumes its maximum over spatial scale $\sigma _1$ and over temporal scale $\sigma _t$ at

130 $$\begin{aligned} \hat{\omega }_{\varphi \varphi ,t} = \frac{\sqrt{2}}{\sigma _1 \sqrt{\cos ^2 \theta + \kappa ^2 \sin ^2 \theta }}, \end{aligned}$$

131 $$\begin{aligned} \hat{u}_{\varphi \varphi ,t} = \frac{\sigma _1}{\sqrt{2}\sigma _t} \sqrt{\cos ^2 \theta + \kappa ^2 \sin ^2 \theta }, \end{aligned}$$

and again reparameterizing the other spatial scale parameter $\sigma _2$ as $\sigma _2 = \kappa \, \sigma _1$, gives that the maximum amplitude measure over spatial and temporal scales is

132 $$\begin{aligned} A_{\varphi \varphi ,t,\max }(\theta ;\; \kappa ) = \frac{2 \cos ^2 \theta }{e^{3/2} \left( \cos ^2 \theta + \kappa ^2 \sin ^2\theta \right) }. \end{aligned}$$

#### A.1.4 Second-order second-order simple cell

Consider a space-time separable receptive field corresponding to a *second-order* scale-normalized Gaussian derivative with scale parameter $\sigma _1$ in the horizontal $x_1$-direction, a zero-order Gaussian kernel with scale parameter $\sigma _2$ in the vertical $x_2$-direction, and a *second-order* scale-normalized Gaussian derivative with scale parameter $\sigma _t$ in the temporal direction, corresponding to $\varphi = 0$, $v = 0$, $\varSigma _0 = \operatorname {diag}(\sigma _1^2, \sigma _2^2)$, $m = 2$ and $n = 2$ in Eq. (13):

133 $$\begin{aligned} {\begin{matrix}&T_{00,tt,\text{ norm }}(x_1, x_2, t;\; \sigma _1, \sigma _2, \sigma _t) = \end{matrix}}\nonumber \\ {\begin{matrix}&= \frac{\sigma _1^2 \sigma _t^2}{(2 \pi )^{3/2} \, \sigma _1 \sigma _2 \sigma _t} \, \partial _{x_1 x_1} \partial _{tt} \left( e^{-x_1^2/2\sigma _1^2 - x_2^2/2 \sigma _2^2 - t^2/2\sigma _t^2} \right) \end{matrix}}\nonumber \\ {\begin{matrix}&= \frac{(x_1^2 - \sigma _1^2) (t^2 - \sigma _t^2)}{(2 \pi )^{3/2} \, \sigma _1^3 \sigma _2 \sigma _t^3} \, e^{-x_1^2/2\sigma _1^2 - x_2^2/2 \sigma _2^2 - t^2/2\sigma _t^2}. \end{matrix}} \end{aligned}$$

The corresponding receptive field response is then, after solving the convolution integral in Mathematica,

134 $$\begin{aligned} {\begin{matrix} L_{00,tt,\text{ norm }}(x_1, x_2, t;\; \sigma _1, \sigma _2, \sigma _t) = \end{matrix}}\nonumber \\ {\begin{matrix}&= \int _{\xi _1 = -\infty }^{\infty } \int _{\xi _2 = -\infty }^{\infty } \int _{\zeta = -\infty }^{\infty } T_{00,tt,\text{ norm }}(\xi _1, \xi _2, \zeta ;\; \sigma _1, \sigma _2, \sigma _t) \end{matrix}}\nonumber \\ {\begin{matrix}&{\hspace{85.0pt}} \times f(x_1 - \xi _1, x_2 - \xi _2, t - \zeta ) \, d \xi _1 \xi _2 d\zeta \end{matrix}}\nonumber \\ {\begin{matrix}&= \omega ^4 \, \sigma _1^2 \, \sigma _t^2 \, u^2 \cos ^2 (\theta ) \, e^{-\frac{1}{2} \omega ^2 (\sigma _1^2 \cos ^2 \theta + \sigma _2^2 \sin ^2 \theta + \sigma _t^2 u^2)} \end{matrix}}\nonumber \\ {\begin{matrix}&{\hspace{15.0pt}} \times \sin \left( \omega \cos (\theta ) \, x_1 + \omega \sin (\theta ) \, x_2 - u \, t + \beta \right) , \end{matrix}} \end{aligned}$$

*i.e.*, a sine wave with amplitude

135 $$\begin{aligned} A_{\varphi \varphi ,tt}(\theta , u, \omega ;\; \sigma _1, \sigma _2, \sigma _t) = \\ = \omega ^4 \, \sigma _1^2 \, \sigma _t^2 \, u^2 \cos ^2 \theta \, e^{-\frac{1}{2} \omega ^2 (\sigma _1^2 \cos ^2 \theta + \sigma _2^2 \sin ^2 \theta + \sigma _t^2 u^2)}. \end{aligned}$$

This entity assumes its maximum over spatial scale $\sigma _1$ and over temporal scale $\sigma _t$ at

136 $$\begin{aligned} \hat{\omega }_{\varphi \varphi ,tt} = \frac{\sqrt{2}}{\sigma _1 \sqrt{\cos ^2 \theta + \kappa ^2 \sin ^2 \theta }}, \end{aligned}$$

137 $$\begin{aligned} \hat{u}_{\varphi \varphi ,tt} = \frac{\sigma _1}{\sigma _t} \sqrt{\cos ^2 \theta + \kappa ^2 \sin ^2 \theta }, \end{aligned}$$

and again reparameterizing the other spatial scale parameter $\sigma _2$ as $\sigma _2 = \kappa \, \sigma _1$, gives that the maximum amplitude measure over spatial and temporal scales is

138 $$\begin{aligned} A_{\varphi \varphi ,tt,\max }(\theta ;\; \kappa ) = \frac{4 \cos ^2 \theta }{e^2 \left( \cos ^2 \theta + \kappa ^2 \sin ^2 \theta \right) }. \end{aligned}$$

The middle column in Fig. 17 shows the result of plotting the measure $A_{\varphi \varphi ,tt,\max }(\theta ;\; \kappa )$ of the orientation selectivity as function of the inclination angle $\theta$ for a few values of the scale parameter ratio $\kappa$, with the values rescaled such that the peak value is equal to 1. Again, the degree of orientation selectivity increases strongly with the value of $\kappa$, as for the first-order first-order model of a simple cell.

#### A.1.5 Complex cell

To model the spatio-temporal response of a complex cell according to the directional sensitive spatio-temporal quasi-quadrature measure Eq. (13) based on space-time separable spatio-temporal receptive fields, we combine the responses of the first-order first-order simple cell in Eq. (116), the first-order second-order cell in Eq. (122), the second-order first-order simple cell in Eq. (128) and the second-order second order cell in Eq. (134) for $v = 0$, $\varGamma _{\varphi } = 0$ and $\varGamma _t = 0$ according to

139 $$\begin{aligned} {\begin{matrix}&(\mathcal{Q}_{0,\text{ sep },\text{ norm }} L)^2 \end{matrix}}\nonumber \\ {\begin{matrix}&= L_{0,t,\text{ norm }}^2 + C_{\varphi } \, L_{00,t,norm}^2 + \end{matrix}}\nonumber \\ {\begin{matrix}&{\hspace{10.0pt}} + C_t \left( L_{0,tt,\text{ norm }}^2 + C_{\varphi } \, L_{00,tt,\text{ norm }}^2 \right) . \end{matrix}} \end{aligned}$$

Selecting the angular frequency $\hat{\omega }$ as the geometric average of the angular frequencies where the above spatio-temporal simple cell models assume their maximum amplitude responses over spatial scales

140 $$\begin{aligned} \hat{\omega }_\mathcal{Q} = \sqrt{\hat{\omega }_{\varphi ,t} \, \hat{\omega }_{\varphi ,tt} \, \hat{\omega }_{\varphi \varphi ,t} \, \hat{\omega }_{\varphi \varphi ,tt}} = \frac{\root 4 \of {2}}{\sigma _1 \sqrt{\cos ^2 \theta + \kappa ^2 \sin ^2 \theta }}, \end{aligned}$$

and selecting the image velocity $\hat{u}$ of the sine wave as the geometric average of the image velocities where the above spatio-temporal simple cell models assume their maximum amplitude responses over image velocities

141 $$\begin{aligned} \hat{u}_\mathcal{Q} = \sqrt{\hat{u}_{\varphi ,t} \, \hat{u}_{\varphi ,tt} \, \hat{u}_{\varphi \varphi ,t} \, \hat{u}_{\varphi \varphi ,tt}} = \frac{\sigma _1}{\sigma _t} \sqrt{\cos ^2 \theta + \kappa ^2 \sin ^2 \theta }, \end{aligned}$$

as well as choosing the spatial and temporal weighting factors $C_{\varphi }$ and $C_t$ between first- and second-order information as $C_{\varphi } = 1/\sqrt{2}$ and $C_t = 1/\sqrt{2}$ according to Lindeberg (2018), then implies that the spatio-temporal quasi-quadrature measure assumes the form

142 $$\begin{aligned} A_{\mathcal{Q},\text{ sep }}(\theta ;\; \kappa ) = \frac{e^{-\sqrt{2}} \left| \cos \theta \right| \, \sqrt{2 + \kappa ^2 + (2 - \kappa ^2) \cos 2 \theta }}{\cos ^2\theta + \kappa ^2 \sin ^2\theta }. \end{aligned}$$

Note that this expression is independent of both the spatial scale parameter $\sigma _1$ and the temporal scale parameter $\sigma _t$, because of the scale-invariant properties of scale-normalized derivatives for scale normalization parameter $\gamma = 1$. Moreover, this expression is also independent of the phase of the signal, as determined by the spatial coordinates $x_1$ and $x_2$, the time moment *t* and the phase angle $\beta$.

The right column in Fig. 17 shows the result of plotting the measure $A_{\mathcal{Q},\text{ sep }}(\theta ;\; \kappa )$ of the orientation selectivity as function of the inclination angle $\theta$ for a few values of the scale parameter ratio $\kappa$, with the values rescaled such that the peak value for each graph is equal to 1. As can be seen from the graphs, the degree of orientation selectivity increases strongly with the value of $\kappa$ also for this spatio-temporal model of a complex cell, and in a qualitatively similar way as for the simple cell models, regarding both the purely spatial as well as the joint spatio-temporal models of the simple cells.

### A.2 Affine transformation property for receptive fields according to a generalized affine Gabor model for simple cells

Given any positive definite spatial covariance matrix $\varSigma$ and any angular frequency vector $\omega = (\omega _1, \omega _2)^T$, consider a generalization of the affine Gabor model in Eq. (61) to a complex affine Gabor function of the form

143 $$\begin{aligned} G(x;\; \varSigma , \omega ) = g(x;\; \varSigma , \omega ) \, e^{i \omega ^T x}, \end{aligned}$$

where $g(x;\; \varSigma , \omega )$ denotes a (here) two-dimensional Gaussian kernel

144 $$\begin{aligned} g(x;\; \varSigma ) = \frac{1}{2 \pi \sqrt{\det \varSigma }} \, e^{-x^T \varSigma ^{-1} x/2}. \end{aligned}$$

In the case when the angular frequency vector $\omega$ is parallel to one of the eigenvectors of the spatial covariance matrix $\varSigma$, this form of affine Gabor function spans a similar variability as the previously treated affine Gabor model in Eq. (61). To make it possible to define a representation that is closed under general affine transformations (affine covariance), we do, however, here, relax the assumption that the orientation of the angular frequency vector $\omega$ should be related to the dominant orientations defined by the eigendirections of the spatial covariance matrix $\varSigma$.

Let us now assume that we have two spatial images *f*(*x*) and $f'(x')$ that are related according to a spatial affine transformation, such that

145 $$\begin{aligned} f'(x') = f(x) \end{aligned}$$

for

146 $$\begin{aligned} x' = A \, x. \end{aligned}$$

Let us furthermore define affine Gabor representations over the two spatial domains according to

147 $$\begin{aligned} {\begin{matrix} L(x;\, \varSigma , \omega )&= (G(\cdot ;\; \varSigma , \omega ) * f(\cdot ))(x;\; \varSigma , \omega ) \end{matrix}}\nonumber \\ {\begin{matrix}&= \int _{\xi \in {\mathbb {R}}^2} G(\xi ;\; \varSigma , \omega ) \, f(x - \xi ) \, d\xi , \end{matrix}}\end{aligned}$$

148 $$\begin{aligned} {\begin{matrix} L'(x';\, \varSigma ', \omega ')&= (G(\cdot ;\; \varSigma ', \omega ') * f'(\cdot ))(x';\; \varSigma ', \omega ') \end{matrix}}\nonumber \\ {\begin{matrix}&= \int _{\xi ' \in {\mathbb {R}}^2} G(\xi ';\; \varSigma ', \omega ') \, f(x' - \xi ') \, d\xi '. \end{matrix}} \end{aligned}$$

We would now like to investigate if the affine Gabor representations over the two domains could be related to each other. To express such a relation, we will perform the change of variables

149 $$\begin{aligned} \xi ' = A \, \xi , \end{aligned}$$

which gives

150 $$\begin{aligned} d\xi ' = |\det A| \, d\xi , \end{aligned}$$

in the convolution integrals.

**Step I:** Let us first consider how the Gaussian function $g'(x';\, \varSigma ')$ in the Gabor function $G(x';\; \varSigma ', \omega ')$ transforms under the change of variables. If we, in analogy with the transformation property of the spatial covariance matrix in the affine Gaussian derivative model for visual receptive fields (Equation (28) in Lindeberg 2023b) assume that

151 $$\begin{aligned} \varSigma ' = A \, \varSigma \, A^T, \end{aligned}$$

which gives

152 $$\begin{aligned} {\varSigma ' }^{-1}= A^{-T} \, \varSigma ^{-1} \, A^{-1} \end{aligned}$$

and

153 $$\begin{aligned} \det \varSigma ' = | \det A |^2 \, \det \varSigma , \end{aligned}$$

then we obtain

154 $$\begin{aligned} {\begin{matrix} g(\xi ';\; \varSigma ') = \frac{1}{2 \pi \sqrt{\det \varSigma '}} \, e^{-{\xi '}^T {\varSigma '}^{-1} \xi '/2} \end{matrix}}\nonumber \\ {\begin{matrix} = \frac{1}{2 \pi | \det A | \sqrt{\det \varSigma }} \, e^{-{A \xi }^T A^{-T} \, \varSigma ^{-1} \, A^{-1} A \xi /2} \end{matrix}}\end{aligned}$$

155 $$\begin{aligned} {\begin{matrix} = \frac{1}{| \det A |} \, \frac{1}{2 \pi \sqrt{\det \varSigma }} \, e^{-\xi ^T\, \varSigma ^{-1} \xi /2} = \frac{1}{| \det A |} \, g(\xi ;\; \varSigma ). \end{matrix}} \end{aligned}$$

**Step II:** If we further set

156 $$\begin{aligned} \omega ' = A^{-T} \omega , \end{aligned}$$

and combine with the transformation property in Eq. (155) of the affine Gaussian kernel, then we have that the Gabor functions over the two domains are related according to

157 $$\begin{aligned} {\begin{matrix} & G(\xi ';\; \varSigma ', \omega ') = g(\xi ';\; \varSigma ') \, e^{i {\omega '}^T \xi '}\\ & \quad = \frac{1}{| \det A |} \, g(\xi ;\; \varSigma ) \, e^{i (A^{-T} \omega )^T A \xi } \end{matrix}}\nonumber \\ {\begin{matrix}&\quad = \frac{1}{| \det A |} \, g(\xi ;\; \varSigma ) \, e^{i \omega ^T \xi } = \frac{1}{| \det A |} \, G(\xi ;\; \varSigma , \omega ). \end{matrix}} \end{aligned}$$

**Step III:** Applied to the convolution integrals in Eqs. (147) and (148), the transformation property in Eq. (157) of the genuinely affine Gabor function in Eq. (143) thus implies that

158 $$\begin{aligned} {\begin{matrix} L'(x';\, \varSigma ', \omega ') = (G(\cdot ;\; \varSigma ', \omega ') * f'(\cdot ))(x';\; \varSigma ', \omega ') \end{matrix}}\nonumber \\ {\begin{matrix} = \int _{\xi ' \in {\mathbb {R}}^2} G(\xi ';\; \varSigma ', \omega ') \, f(x' - \xi ') \, d\xi '. \end{matrix}}\nonumber \\ {\begin{matrix} = \int _{\xi \in {\mathbb {R}}^2} \frac{1}{| \det A |} \, G(\xi ;\; \varSigma , \omega ) \, f(x - \xi ) \, | \det A | \, d\xi , \end{matrix}}\nonumber \\ {\begin{matrix} = (G(\cdot ;\; \varSigma , \omega ) * f(\cdot ))(x;\; \varSigma , \omega ) = L(x;\, \varSigma , \omega ). \end{matrix}} \end{aligned}$$

#### Summary of main result

To conclude, we have shown that if two images *f*(*x*) and $f'(x')$ are related according to an affine transformation

159 $$\begin{aligned} f'(x') = f(x) \end{aligned}$$

where

160 $$\begin{aligned} x' = A \, x, \end{aligned}$$

then provided that affine Gabor representations are defined over the two spatial domains according to

161 $$\begin{aligned} {\begin{matrix} L(x;\, \varSigma , \omega )&= (G(\cdot ;\; \varSigma , \omega ) * f(\cdot ))(x;\; \varSigma , \omega ), \end{matrix}}\end{aligned}$$

162 $$\begin{aligned} {\begin{matrix} L'(x';\, \varSigma ', \omega ')&= (G(\cdot ;\; \varSigma ', \omega ') * f'(\cdot ))(x';\; \varSigma ', \omega '), \end{matrix}} \end{aligned}$$

it then follows that these (genuinely) affine Gabor representations are equal under a similar spatial affine transformation

163 $$\begin{aligned} L'(x';\, \varSigma ', \omega ') = L(x;\, \varSigma , \omega ), \end{aligned}$$

provided that the values of the receptive field parameters $\varSigma$, $\varSigma '$, $\omega$ and $\omega '$ are appropriately matched according to

164 $$\begin{aligned} {\begin{matrix} \varSigma ' = A \, \varSigma \, A^T, \end{matrix}}\end{aligned}$$

165 $$\begin{aligned} {\begin{matrix} \omega ' = A^{-T} \omega . \end{matrix}} \end{aligned}$$

This affine transformation property constitutes a genuine affine covariance property of the (here) generalized family of affine Gabor functions according to

166 $$\begin{aligned} G(x;\; \varSigma , \omega ) = g(x;\; \varSigma , \omega ) \, e^{i \omega ^T x}. \end{aligned}$$

In this context, it should, however, be noted that restricted affine Gabor model Eq. (61), where the angular frequency vector is restricted to be parallel with one of the eigendirections of the spatial covariance matrix $\varSigma$, cannot be expected to be covariant over the complete group of spatial affine transformations. That restricted affine Gabor model will, however, be covariant under similarity transformations (uniform scaling transformations and spatial rotations) as well as to stretching transformations in the orientation of the angular frequency vector.

## Footnotes

1 Notes concerning the terminology used in this paper: While the notion of a visual receptive field originally referred to the region in the visual field that may invoke a stimulus response for a visual neuron Hubel and Wiesel (1959, 2005), we here adopt a somewhat different functional definition of the notion of a receptive field, as the visual operator that computes the output from a visual neuron for a (non-spiking) model of the computational function of the visual neuron. From such a viewpoint, the above more traditional notion of a visual receptive field is according to the definition used in this paper instead referred to as the (effective) support region of the receptive field. Concerning simple cells, we throughout this paper make use of idealized purely feed-forward computational models of such receptive fields in terms of linear convolution operations, whereas our idealized models of complex cells do instead combine the output from such linear simple cells in a non-linear, also purely feed-forward, manner. These highly idealized simplifications are made here to make theoretical analysis feasible to handle in closed form, thereby leading to a set of closed-form characteristics regarding the orientation selectivity properties for the two main paradigms of modelling simple and complex cells in terms of either Gaussian derivative operators or Gabor functions.

2 If we let $e_{\varphi }$ denote the unit vector in the direction of $\varphi$, according to $e_{\varphi } = (\cos \varphi , \sin \varphi )^T$, then we can determine $\sigma _{\varphi }$ from the spatial covariance matrix $\varSigma _{\varphi }$ according to $\sigma _{\varphi } = \sqrt{e_{\varphi }^T \varSigma _{\varphi } \, e_{\varphi }}$.

3 In this paper, we parameterize both the spatial and the temporal scale parameters in units of the standard deviations of the corresponding spatial or temporal smoothing kernels. These parameters are related to the corresponding variance-based parameterizations in the earlier work, that we build upon, according to $\sigma _{\text{ space }} = \sqrt{s}$ and $\sigma _{\text{ time }} = \sqrt{\tau }$. By this change of notation, some equations in this paper will have somewhat different appearance, compared to the previous work that we cite. This change of notation will, however, lead to easier notation in the mathematical analysis that will follow in Sect. 4

4 Concerning the notation, we write derivatives either in operator form $\partial _{x_1}$, which constitutes short notation for $\frac{\partial }{\partial x_1}$, or as subscripts, $L_{x_1} = \partial _{x_1} L$, which is also short notation for $\frac{\partial L}{\partial x_1}$. Directional derivatives of functions are also written as subscripts $L_{\varphi } = \partial _{\varphi } L$ and can, of course, also be applied repeatedly $L_{\varphi \varphi } = \partial _{\varphi }^2 L$. Observe, however, that subscripts are also used with a different meaning in connections constants. The notation $\sigma _{\varphi }$ means the $\sigma$-value used when computing directional derivatives, as opposed to $\sigma _t$, to be introduced later, which means the $\sigma$-value that is used when computing temporal derivatives.

5 In the expressions below, the symbol *L* denotes the result of pure spatial smoothing of the input image $f(x_1, x_2)$ with an affine Gaussian kernel $g(x_1, x_2;\; \varSigma )$, *i.e.* $L(x_1, x_2;\; \varSigma ) = g(x_1, x_2;\; \varSigma ) * f(x_1, x_2)$. In the area of scale-space theory, this representation is referred to as the (affine) spatial scale-space representation of *f*.

6 Note, however, that, since the input signals in this analysis are throughout sine waves, we could also get the receptive field responses to the sine waves by multiplying the amplitudes of the sine waves by the absolute values of the Fourier transforms of the receptive field models, while simultaneously shifting the phases of the sine waves with the phases of the Fourier transforms of the receptive field models. For convenience, we do, however, throughout this treatment solve the convolution integrals as explicit integrals in Mathematica, since symbolic computations are really helpful in this mathematical analysis, when optimizing the scale selectivity properties of the receptive fields, by differentiating the receptive field responses with respect to the angular frequencies of the sine waves, and also to handle the more complex non-linear models of complex cells, considered later in this paper.

7 Concerning the choice of the weighting factor $C_{\varphi }$ between first- and second-order information, it holds that $C_{\varphi } = 1/\sqrt{2}$ implies that the spatial quasi-quadrature measure will assume a constant value (be phase independent) for a sine wave at the scale level that is the geometric average of the scale levels at which the scale-normalized amplitudes of the first- and the second-order components in the quasi-quadrature measure assume their maxima over scale, for the specific choice of $\gamma = 1$ and $\varGamma = 0$. We will later see manifestations of this property, in that the responses of the different quasi-quadrature measures, that we use for modelling complex cells, will be phase independent for inclination angle $\theta = 0$, for an angular frequency that is the geometric average of the angular frequencies for which the first- and second-order components in the quasi-quadrature measures will assume their maximum amplitude over scales (see Eqs. (26), (142) and (43)).

8 In the orientation selectivity analysis for receptive fields according to the affine Gabor model in Section 5, we did, however, not optimize the angular frequency $\omega$ of the probing sine wave for each inclination angle $\theta$, because that was harder to accomplish, in terms of closed-form mathematical expressions.
