# Supplementary material for: Orientation selectivity properties for the affine Gaussian derivative and the affine Gabor models for visual receptive fields
Source: J Comput Neurosci. 2025 Jan 29;53(1):61–98. doi: 10.1007/s10827-024-00888-w (PMC11868404; doi:10.1007/s10827-024-00888-w)
Supplement: Supplementary file 1 — (zip 821 KB) [file 10827_2024_888_MOESM1_ESM.zip › JCompNeuroSci-supplementary-files/README.pdf]

README file for Mathematica notebooks for the paper

Lindeberg (2024) "Orientation selectivity properties for the affine Gaussian derivative and the affine Gabor models for visual receptive fields", submitted to Journal of Computational Neuroscience.

Regarding the different sections in the paper, the closed-form calculations are performed in the following Mathematica notebooks:

Section 4.3: Analysis of *purely spatial* generalized Gaussian derivative based receptive fields

affgaussRF-ori-sel.nb

Section 4.4 and Appendix A.1: Analysis of *space-time-separable spatio-temporal* generalized Gaussian derivative based receptive fields

space-time-sepRF-ori-sel.nb

Section 4.5: Analysis of *velocity-adapted spatio-temporal* generalized Gaussian derivative based receptive fields

space-time-nonsepRF-ori-sel.nb

Section 5: Analysis of the *purely spatial* affine Gabor model

ori-sel-gabor.nb

Sections 6 and 7.2.1: Compact descriptors of orientation selectivity curves

compact-ori-sel-measures.nb

Sections 7.1-7.2: Perturbation analysis with regard to the tuning of the sine wave probes to the receptive field structure for the *purely spatial* generalized Gaussian derivative based receptive fields

affgaussRF-ori-sel-perturb.nb

Sections 7.3-7.4: Perturbation analysis with regard to the tuning of the sine wave probes to the receptive field structure for the *velocity-adapted spatio-temporal* generalized Gaussian derivative based receptive fields

space-time-nonsepRF-ori-sel-perturb.nb
